# Small-molecule sequestration of amyloid-β as a drug discovery strategy for Alzheimer’s disease

**DOI:** 10.1126/sciadv.abb5924

**Published:** 2020-11-04

**Authors:** Gabriella T. Heller, Francesco A. Aprile, Thomas C. T. Michaels, Ryan Limbocker, Michele Perni, Francesco Simone Ruggeri, Benedetta Mannini, Thomas Löhr, Massimiliano Bonomi, Carlo Camilloni, Alfonso De Simone, Isabella C. Felli, Roberta Pierattelli, Tuomas P. J. Knowles, Christopher M. Dobson, Michele Vendruscolo

**Affiliations:** 1 Centre for Misfolding Diseases, Department of Chemistry, University of Cambridge, Cambridge CB2 1EW, UK.; 2 Department of Chemistry, Molecular Sciences Research Hub, Imperial College London, London W12 0BZ, UK.; 3 Paulson School of Engineering and Applied Sciences, Harvard University, Cambridge, MA, USA.; 4 Structural Bioinformatics Unit, Department of Structural Biology and Chemistry. CNRS UMR 3528, C3BI, CNRS USR 3756, Institut Pasteur, Paris, France.; 5 Dipartimento di Bioscienze, Università degli Studi di Milano, 20133 Milano, Italy.; 6 Division of Molecular Biosciences, Imperial College London, London SW7 2AZ, UK.; 7 Department of Pharmacy, University of Naples “Federico II,” 80131 Naples, Italy.; 8 Magnetic Resonance Center (CERM), University of Florence, 50019 Sesto Fiorentino, Italy.; 9 Department of Chemistry “Ugo Schiff,” University of Florence, 50019 Sesto Fiorentino, Italy.

## Abstract

Disordered proteins are challenging therapeutic targets, and no drug is currently in clinical use that modifies the properties of their monomeric states. Here, we identify a small molecule (10074-G5) capable of binding and sequestering the intrinsically disordered amyloid-β (Aβ) peptide in its monomeric, soluble state. Our analysis reveals that this compound interacts with Aβ and inhibits both the primary and secondary nucleation pathways in its aggregation process. We characterize this interaction using biophysical experiments and integrative structural ensemble determination methods. We observe that this molecule increases the conformational entropy of monomeric Aβ while decreasing its hydrophobic surface area. We also show that it rescues a *Caenorhabditis elegans* model of Aβ-associated toxicity, consistent with the mechanism of action identified from the in silico and in vitro studies. These results illustrate the strategy of stabilizing the monomeric states of disordered proteins with small molecules to alter their behavior for therapeutic purposes.

## Teaser

A small molecule binds to a disordered protein in its monomeric form, preventing its aggregation linked to Alzheimer’s disease.

## INTRODUCTION

Alzheimer’s disease is a fatal neurodegenerative condition and the leading cause of dementia, which affects more than 50 million people worldwide, a number that is predicted to rise to 150 million by 2050 unless methods of prevention or treatment are found (*1*). Despite over 25 years of intensive research and hundreds of clinical trials, there is still no drug capable of modifying the course of this disease (*1*).

The aggregation of the amyloid-β (Aβ) peptide in brain tissue is one of the hallmarks of Alzheimer’s disease (*2*). This process involves at least three forms of Aβ: (i) a monomeric state, which is highly disordered; (ii) oligomeric aggregates, which are heterogeneous, transient, and cytotoxic; and (iii) fibrillar structures, which are ordered and relatively inert, although they are capable of catalyzing the formation of Aβ oligomers (*3*). More generally, the aggregation of Aβ involves a complex nonlinear network of interdependent microscopic processes, including (i) primary nucleation, in which oligomers form from monomeric species; (ii) elongation, in which oligomers and fibrils increase in size by monomer addition; (iii) secondary nucleation, whereby the surfaces of fibrillar aggregates catalyze the formation of new oligomeric species; and (iv) fragmentation, in which fibrils break into smaller pieces, increasing the total number of oligomers and fibrils capable of elongation (*2*).

Aβ is produced by proteolysis from the transmembrane amyloid precursor protein, and its 42-residue form (Aβ42) is the predominant species in deposits characteristically observed in the brains of patients with Alzheimer’s disease (*4*). Kinetic analysis shows that, once a critical concentration of Aβ42 fibrils has been formed, secondary nucleation overtakes primary nucleation in becoming the major source of Aβ42 oligomers, as fibril surfaces act as catalytic sites for their formation (*3*). The fact that the oligomers appear to be the most toxic species formed during the aggregation process (*5*, *6*), however, suggests that therapeutic strategies targeting Aβ aggregation should not primarily aim at inhibiting fibril formation per se, but rather doing so in a manner that specifically reduces the generation of oligomeric species (*2*). Complex feedback mechanisms between the different microscopic steps in the aggregation reaction can lead to an increase in the concentration of oligomers even when the formation of fibrils is inhibited and hence result in an increase in pathogenicity (*2*).

Previous studies have suggested that effective strategies for inhibiting Aβ aggregation could be based on targeting fibril surfaces to suppress the generation of oligomers or the reduction of the toxicity of the oligomers (*7*–*11*). It is unclear, however, whether sequestering Aβ in its soluble state could be an effective drug discovery strategy against Alzheimer’s disease. Stabilization of monomeric Aβ into a β-hairpin conformation with large biomolecules has been previously demonstrated to inhibit aggregation, for example, using an affibody protein (*12*). However, whether this stabilization of Aβ in its monomeric form can be achieved via small-molecule binding in a drug-like manner is still under debate. While there is research indicating a stabilizing effect of small molecules on the soluble state of Aβ, there are contradictory reports of their effects on its aggregation (*13*–*15*). It should also be considered that these molecules may not be specific, as for example, some appear to bind monomeric Aβ in a manner similar to low concentrations of SDS (*13*–*15*). Furthermore, it has been proposed that the binding of these small molecules to monomeric Aβ may be mediated by colloidal particles formed by the small molecules (*16*), although this observation has also been disputed (*13*, *14*). The uncertainty of whether monomeric Aβ is a viable drug target is caused, in part, by a lack of understanding of the molecular properties of monomeric Aβ and how to stabilize this peptide with specific small molecules that have the potential to be developed as drugs.

Aβ, in its monomeric form, is intrinsically disordered, as it lacks a well-defined structure and instead exists as a heterogeneous ensemble of conformationally distinct states (*17*). The dynamic nature of disordered proteins, and the consequent absence of stable and persistent binding pockets, implies that they do not readily lend themselves to conventional, enthalpy focused mechanisms of drug binding, such as the well-established lock-and-key paradigm, in which a drug fits snugly into a single, well-defined binding site (*18*, *19*). As a result, targeting disordered proteins with small molecules has not been considered a promising drug discovery strategy, and there are no small molecules on the market directly targeting disordered regions despite their high prevalence in disease. Recently, it has been proposed that it may be possible to bind disordered proteins with small molecules via the entropic expansion mechanism, in which the conformational entropy of the disordered protein increases upon binding a small molecule (*18*), although until now, this has yet to be demonstrated. A deeper understanding of the possible mechanisms by which small molecules can modify the behavior of disordered proteins may open new avenues for drug development, not only against Alzheimer’s disease and other neurodegenerative disorders but also many other medical conditions involving disordered proteins, including type 2 diabetes, certain forms of cancer, and cardiovascular disease (*17*, *18*). This insight may also be particularly valuable in modulating liquid-liquid phase separation, which often involves protein disorder (*20*).

**Fig. 1:**
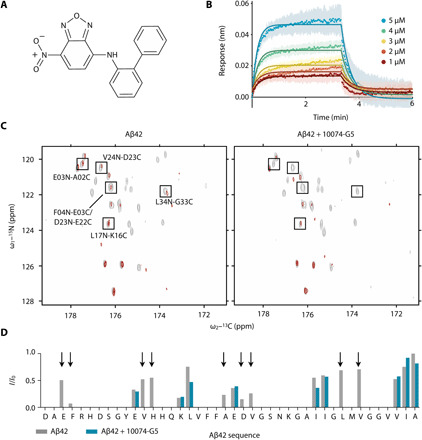
Characterization of the interaction of 10074-G5 with monomeric Aβ42. (**A**) Structure of biphenyl-2-yl-(7-nitro-benzo[1,2,5]oxadiazol-4-yl)-amine, also known as 10074-G5. (**B**) Biolayer interferometry measurements showing the dose-dependent binding of 10074-G5 to an Aβ42-functionalized surface at various concentrations of the added compound. The curves were corrected for baseline drift. Raw data are shown in fig. S1A. Control curves showing negligible nonspecific binding are shown in fig. S1 (B and D). Global fitting to simple one-phase association and dissociation equations yields association (*k*_on_) and dissociation (*k*_off_) rates to be 8.5 × 10^3^ ± 0.2 × 10^3^ M^−1^ s^−1^ and 4.7 × 10^−2^ ± 2 × 10^−4^ s^−1^, respectively, corresponding to a binding dissociation constant (*K*_d_) of 6 μM. For this fit, all five curves were constrained to single, shared *k*_on_ and *k*_off_ values. The global *R*^2^ for the fits is 0.98. (**C**) 2D H^N–BEST^CON spectra in the absence (left) and presence (right) of 1:2 Aβ42:10074-G5 with (red) and without (gray) selective water presaturation, performed at 15°C. (**D**) Quantification of the relative *I*/*I*_0_ intensities from (C) shows that the peptide amide groups are more exposed to solvent in the presence of 10074-G5 (blue) as compared to its absence (gray). Arrows highlight regions along the sequence in which signals are detectable in the absence of the compound, but not in its presence, thus suggesting that 10074-G5 increases the solvent exposure of specific regions of Aβ42. ppm, parts per million.

Using experimental and computational biophysical techniques and mathematical modeling, we characterize the interaction of the small-molecule 10074-G5 [biphenyl-2-yl-(7-nitro-benzo[1,2,5]oxadiazol-4-yl)-amine; Fig. 1A], with Aβ42 in its disordered, monomeric state. 10074-G5 has been previously identified to inhibit c-Myc-Max heterodimerization (*21*), specifically by binding and stabilizing the intrinsically disordered c-Myc monomer (*22*, *23*). Here, we also observe that 10074-G5 binds monomeric Aβ42, a disordered peptide unrelated to c-Myc. As a result of this interaction, 10074-G5 significantly delays both primary and secondary nucleation pathways in Aβ42 aggregation. We characterize this interaction using biophysical experiments and integrative structural ensemble determination techniques and observe that Aβ42 remains disordered in the bound form with decreased hydrophobicity. Notably, we also observe that the conformational entropy of Aβ42 increases upon interacting with 10074-G5 via the entropic expansion mechanism, suggesting that exploiting this phenomenon may be a potential therapeutic strategy for disordered proteins. We further show that this molecule inhibits the paralysis associated with Aβ42 aggregation in a *Caenorhabditis elegans* muscle model of Aβ42-mediated toxicity in a manner consistent with the binding mechanism described in silico and characterized in vitro.

## RESULTS

### Selection of the system

We selected the compound 10074-G5 as model system to understand whether and how a small molecule inhibits the aggregation of Aβ by binding the monomeric form of this peptide. We used this molecule as it has been reported to bind another disordered protein in its monomeric form, the oncogenic c-Myc, and it contains a nitrobenzofurazan moiety, which has been previously shown to inhibit the aggregation of Aβ (*24*).

### Characterization of the binding of 10074-G5 to monomeric Aβ42

We characterized the binding of 10074-G5 with monomeric Aβ42 using a multidisciplinary approach based on experiments and integrative structural ensemble determination. First, we carried out biolayer interferometry (BLI; see Materials and Methods) measurements to characterize this interaction in real time. We immobilized N-terminally biotinylated monomeric Aβ42 on the surface of super streptavidin sensor tips (Materials and Methods) and exposed them to varying concentrations of the small molecule (Fig. 1B and fig. S1). We observed a concentration-dependent response, indicative of binding. We globally fit the binding curves to simple one-step association and dissociation equations such that the fit constrains all curves to share single association (*k*_on_) and dissociation (*k*_off_) rates, with a global *R*^2^ (coefficient of determination) value of 0.98. From these fits, we determined *k*_on_ to be 8.5 × 10^3^ ± 0.2 × 10^3^ M^−1^ s^−1^ and *k*_off_ to be 4.7 × 10^−2^ ± 2 × 10^−4^ s^−1^, corresponding to a binding dissociation constant (*K*_d_) of 6 μM. This affinity value is comparable to other small-molecule interactions with disordered proteins (*22*), although we note that constraining disordered monomeric Aβ42 to a surface may perturb the binding that occurs as compared to the unconstrained peptide in solution.

We then investigated the binding of 10074-G5 and monomeric Aβ42 at the ensemble-averaged, single residue level. To do so, we performed 2D H^N–BEST^CON nuclear magnetic resonance (NMR) experiments (*25*) on uniformly ^13^C, ^15^N-labeled monomeric Aβ42 in the presence of one- and twofold concentrations of 10074-G5. As monomeric Aβ42 is relatively stable in solution at low concentrations and temperatures, we examined the binding of 10074-G5 to monomeric Aβ42 under these conditions (20 μM Aβ42 at 5°C). Minimal chemical shift perturbations were observed in the 2D H^N–BEST^CON spectra upon the addition of compound at a 2:1 ligand:protein ratio at 5°C (fig. S2A), consistent with other reports of small-molecule binders of disordered proteins (*22*, *26*), suggesting that Aβ42 remains disordered in the presence of 10074-G5. We then performed this experiment at 15°C in the absence and presence of presaturation of the solvent. This experiment, which relies on heteronuclear direct detection with minimal perturbation of proton polarization, provides a valuable tool to study solvent exposed systems in which amide protons experience fast hydrogen exchange (*25*). In particular, the signals of amide nitrogen atoms become attenuated when their directly bound protons are in fast exchange with the solvent. After testing this on a well-characterized protein (ubiquitin), we performed this experiment on Aβ42 (Fig. 1C). In the absence of 10074-G5, we observed that several hydrophobic residues, particularly C-terminal residues, are protected from solvent exchange (Leu^17^, Leu^34^, Val^36^, Ile^41^, and Ala^42^; see *I*/*I*_0_ values as shown in Fig. 1D). Notably, in the presence of 10074-G5, we observed the quenching of several residues across the sequence of the monomeric Aβ42 peptide that were not quenched in the absence of 10074-G5 (Glu^3^, Phe^4^, Val^12^, His^13^, Ala^21^, Asp^23^, Val^24^, Leu^34^, and Val^36^; see *I*/*I*_0_ values as shown in Fig. 1D), suggesting that some residues have increased solvent exposure in the presence of the small molecule. This change in the solvent exchange profile suggests that 10074-G5 interacts with monomeric Aβ42 in a manner that increases the solubility of at least some of the conformations within the monomeric structural ensemble (*18*).

To obtain further insight into the thermodynamic properties of this interaction, we quantified the heat changes upon 10074-G5 binding to Aβ40 using isothermal titration calorimetry methods (fig. S2, B and C). In these experiments, we used Aβ40 instead of Aβ42 because of the higher solubility of Aβ40; we have, however, shown that 10074-G5 has similar effects on the aggregation of Aβ40 as on that of Aβ42 (fig. S3A). The observation of minimal heat changes (fig. S2, B and C) suggests that the interaction of 10074-G5 with monomeric Aβ has minimal enthalpy and is thus likely to be entropic, as found for the interactions of another small molecule with a disordered peptide (*27*).

**Fig. 2:**
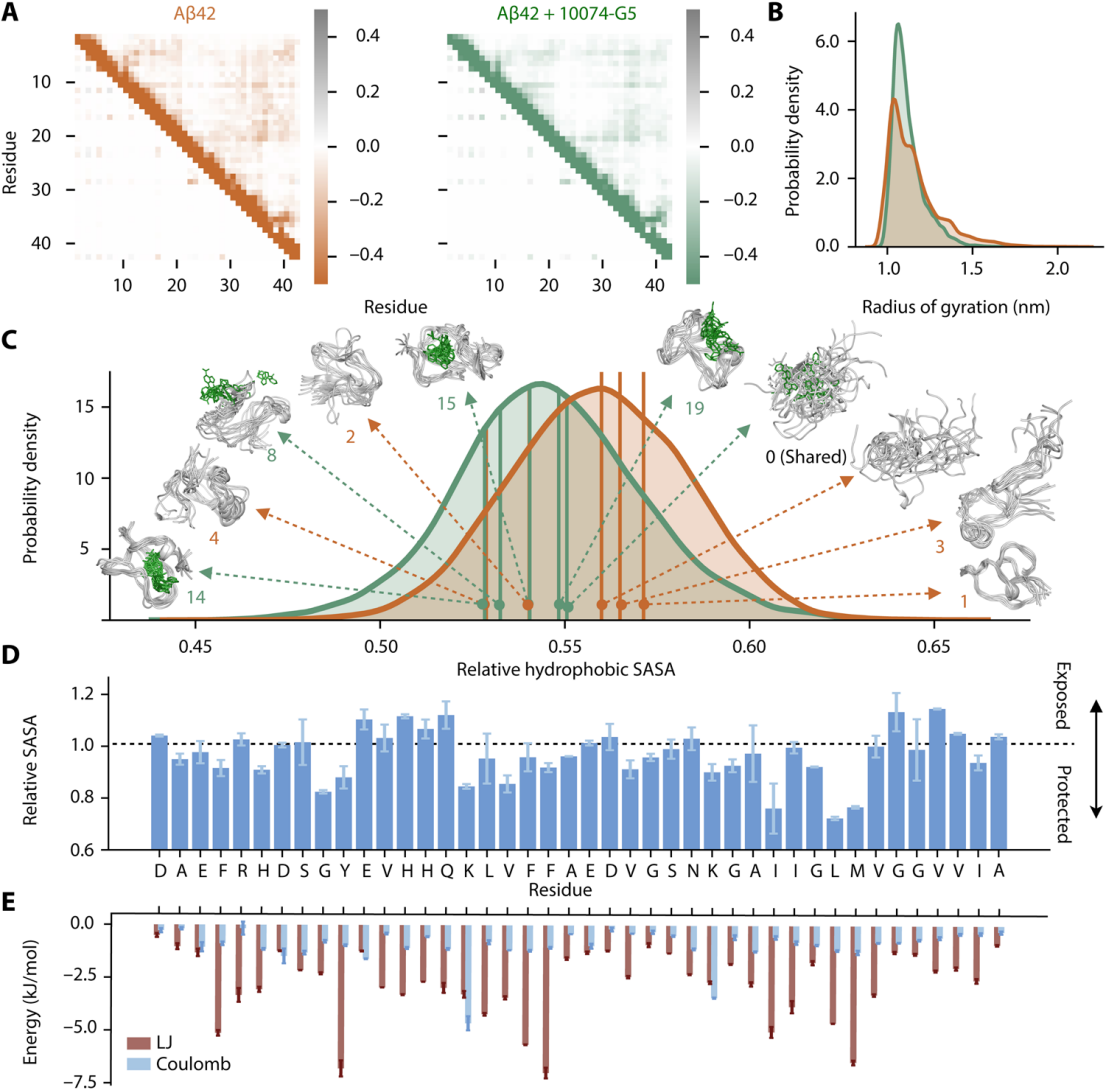
Metadynamic metainference simulations characterize the dynamic binding and show how 10074-G5 promotes Aβ42 conformations with less hydrophobicity. (**A**) Metadynamics metainference simulations demonstrate that inter-residue contact maps for Lennard-Jones (LJ) (top right) and Coulomb (top left) potentials for the unbound (orange) and the bound (green) structural ensembles of Aβ42 with 10074-G5 are highly similar. (**B**) Radii of gyration for the unbound and bound structural ensembles are also highly similar (shown using kernel density estimates of 35,000 points each sampled based on metadynamics weights using a Gaussian kernel). (**C**) Relative hydrophobic solvent accessible surface area (SASA) of Aβ42 (total hydrophobic area over total surface area) of the bound and unbound ensembles, showing that 10074-G5 decreases the relative exposed hydrophobicity of Aβ42. The holo ensemble was calculated only on the protein surface but accounts for the presence of the compound. Data are shown using kernel density estimates as described in (B). Some of the representative structures from these distributions are shown. Numbers indicate cluster IDs shown in Fig. 3. (**D**) Ratio (bound/unbound) of the ensemble-averaged, total SASA per residue showing regions of Aβ42 that become more exposed or protected in the presence of 10074-G5. SASAs of the bound ensemble were calculated on the protein in the presence of 10074-G5. (**E**) Ensemble-averaged, residue-specific LJ and Coulomb interaction energies show that 10074-G5 has strong interactions with aromatic and charged residues. Error bars represent SDs between first and second halves of the analyzed trajectories in (D) and (E).

**Fig. 3:**
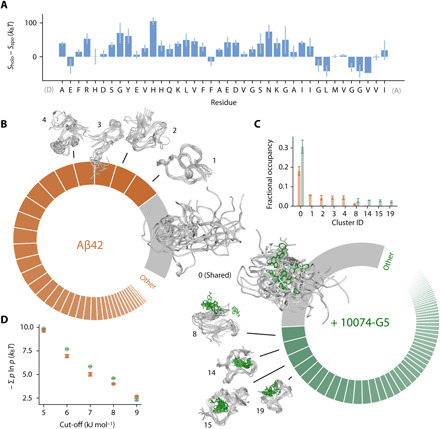
10074-G5 increases the conformational entropy of Aβ42. (**A**) Residue-specific differences in the conformational entropies, *S*, between the holo and apo ensembles, estimated from normalized, two-dimensional (2D) Ramachandran histograms (100 × 100 bins) of each residue using *S* = − Σ *b* ln *b* where *b* is the occupancy of a given bin. (**B**) Donut plots quantifying the conformational states of Aβ42 in the unbound (left, orange) and bound (right, green) simulations. Clustering was performed on concatenated trajectories, considering only Aβ42. Inter-residue contact maps based on the Lennard-Jones potential were used as input for GROMOS clustering (*34*). The cut-off value is 8.5 kJ mol^−1^. Each slice represents a distinct state. The simulations share one major state (cluster 0, gray), which comprises 18 and 31% of the unbound and bound ensembles, respectively. (**C**) Convergence of the five most populated clusters. Bar plot shows fractional cluster occupancies for the unbound (orange) and bound (green) simulations. Fractional cluster occupancies were calculated on 35,000 frames for each concatenated trajectory sampled based on metadynamics weights. (**D**) The conformational entropy of Aβ42, estimated via Gibbs entropy, is consistently higher in the 10074-G5–bound form of the peptide for several clustering cut-off values. The conformational entropy was calculated such that the weights, *P*, of each state correspond to the fractional occupancy as determined by the GROMOS clustering algorithm (*34*). Error bars represent ± SDs of values calculated from the first and second halves of the simulations in (C) and (D).

To obtain a structural description of how 10074-G5 affects the disordered structural ensemble of Aβ42, we used metadynamic metainference, an integrative structural ensemble determination approach (*28*, *29*) that combines all-atom molecular dynamics simulations with NMR chemical shift data to improve force field accuracy (see Materials and Methods, Figs. 2 and 3, and figs. S4 to S6). These simulations reveal that Aβ42 remains disordered in the form bound to 10074-G5, retaining several ensemble-averaged properties. Within this fuzzy interaction (*30*), we noted that most average inter-residue contacts of the unbound peptide remain the same in the bound form (Fig. 2A). Furthermore, the distributions (Fig. 2B) and average values (apo, 1.146 ± 0.002 nm; holo, 1.116 ± 0.004 nm) of the radii of gyration of the bound and unbound forms of the peptide are also highly similar. The presence of 10074-G5 does, however, alter the conformational ensemble of Aβ42, promoting conformations with lower relative hydrophobic surface area (the fraction of accessible hydrophobic surface area with respect to the total accessible surface area; Fig. 2C). To determine which residues became more exposed or protected in the presence of 10074-G5, we compared the average solvent accessible surface areas per residue in the presence and absence of 10074-G5 (Fig. 2D). Notably, we observe that Leu^34^ and Met^35^ become more protected in the presence of the small molecule. Met^35^ has previously been identified as a key residue for attenuation of aggregation, as oxidation has been shown to reduce the lag time of primary nucleation (*31*). These data from the simulation represent distributions and averages of the conformational ensembles. While Fig. 1D experimentally quantifies residue-specific changes in solvent exposure, the result is both ensemble and time averaged over the duration of the measurement and cannot be quantitively compared to residue-specific average solvent accessible surface areas calculated from these equilibrium simulations (Fig. 2D), as these results are not time averaged.

The decrease in the hydrophobic surface area of Aβ42 prompted us to investigate the role of water in the binding interaction. To this end, we calculated the average number of hydrogen bonds formed by water molecules within the hydration shell (all waters within 0.4 nm of Aβ42). We found that water molecules form 3.07509 ± 0.00004 hydrogen bonds on average in the absence of 10074-G5, a number that increases in its presence to 3.0959 ± 0.0005, getting closer to the bulk-like values of 3.44801 ± 0.00002 and 3.44791 ± 0.00002, for apo and holo systems, respectively, under the conditions investigated here; errors are calculated comparing the first and second halves of the trajectories (fig. S6A) (*32*). As expected, we observed little difference between the average number of hydrogen bonds formed by water molecules in the bulk between the apo and holo simulations (fig. S6B). To determine whether or not this binding could be characterized by the release of water molecules upon association, we calculated the average number of water molecules in the hydration shell and show that this value is similar with and without association (fig. S6C).

Given the lack of experimental evidence for enthalpic contributions to the binding, we investigated whether or not we could detect entropic signatures of binding. Previously, changes in conformational entropy have been estimated from probability distributions of structural parameters, such as the radius of gyration (*33*). Given the similarity of the radius of gyration in the bound and unbound forms of the peptide (Fig. 2B), to increase the sensitivity, we applied this approach to the residue-specific Ramachandran plots (Fig. 3A), finding that for the majority of nonterminal residues, the entropy increases in the bound form of the peptide. Nevertheless, quantifying the total change in the conformational entropy of Aβ42 in the presence and absence of 10074-G5 using this approach is challenging given the correlated nature of consecutive dihedral angles. To unequivocally quantify the differences in conformational entropy of the peptide upon binding the small molecule, structurally characterize the unbound and bound conformations of Aβ42, and assess convergence, we performed a clustering analysis (Fig. 3, B and C, and fig. S6D). In particular, considering only Aβ42, we combined the bound and unbound trajectories and grouped them into states based on inter-residue Lennard-Jones (LJ) interaction energies per frame using the GROMOS clustering algorithm (see the Supplementary Materials) (*34*). We observed that while 10074-G5 binds the extended form (state 0) in a nonspecific manner, all other structural clusters show localization of the compound within well-defined pockets of Aβ42 for specific conformations (Figs. 2C and 3B) involving hydrophobic, hydrophilic, charged, and polar residues (fig. S6D). On the basis of the clustering analysis, we calculated the conformational entropy of Aβ42 in the bound and unbound form (see eq. S10 and Fig. 3D). We observed that the conformational entropy of the peptide increased in the bound form, suggesting that the interaction between Aβ42 and 10074-G5 exhibits the entropic expansion mechanism, in which the entropy of the protein contributes favorably to the binding free energy (Fig. 3D) (*18*). Often, drug binding for folded proteins is described in terms of a lock-and-key binding mechanism, in which a protein is constrained by the binding of a small molecule, and therefore the entropy of the protein contributes unfavorably to the free energy of binding. In stark contrast to this mechanism, in the entropic expansion mechanism, the protein becomes more disordered upon interacting with a small molecule, and thus the conformational landscape is expanded upon binding (*18*). The increase in conformational heterogeneity in the bound form of Aβ42 upon binding 10074-G5 may explain the increase in solvation observed by NMR (Fig. 1, C and D). We hypothesize that the increased interconversion between states of Aβ42 in the presence of 10074-G5 may result in a net exposure of the peptide to the solvent. Investigating this possibility, however, would entail further characterization, which is currently beyond the scope of the simulations presented here; as in the metadynamics framework, we sacrifice dynamic information for improved sampling. Nevertheless, the observation of entropic expansion suggests that the identification of small molecules that increase the conformational entropy of the disordered proteins may be a promising therapeutic strategy for disordered proteins involved in aggregation.

To probe the energetic contributions to this interaction on an ensemble-averaged, residue-specific level, we analyzed Lennard-Jones and Coulomb contributions between 10074-G5 and each residue. We observe strong Lennard-Jones interactions, particularly between aromatic residues (Fig. 2E) Tyr^10^, Phe^19^ and Phe ^20^, and 10074-G5. The strongest Coulomb interactions occur at charged residues Lys^16^ and Lys^28^ (Fig. 2E).

### The small-molecule 10074-G5 sequesters monomeric Aβ42 and inhibits its aggregation

**Fig. 4:**
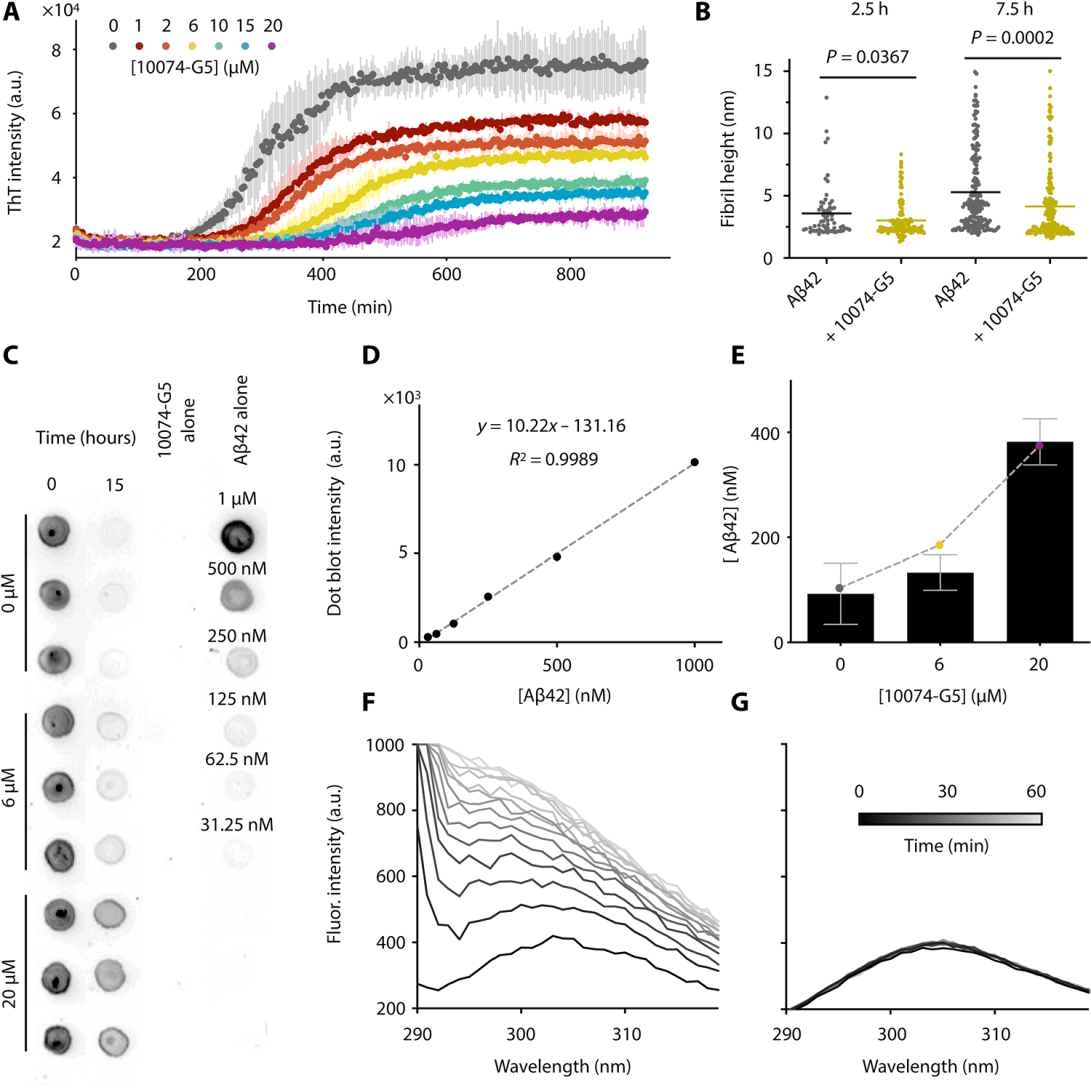
10074-G5 sequesters monomeric Aβ42 and inhibits its aggregation. (**A**) ThT measurements using 1 μM Aβ42 show concentration-dependent effects of 10074-G5 on Aβ42 aggregation. Measurements were taken in triplicate. The concentration of DMSO was held constant across all samples. (**B**) Distributions of cross-sectional heights of 1 μM Aβ42 fibrils at 2.5 hours (*n* ≥ 60) and 7.5 hours (*n* ≥ 200) formed with and without 6 μM 10074-G5, from single-molecule analyses of AFM maps (fig. S7). Lines indicate means. *P* values were determined by unpaired, two-tailed Student’s *t* test. Fibrillar aggregates formed in the presence of 10074-G5 have smaller cross-sectional diameters than those formed in its absence. (**C**) Dot blot of soluble Aβ42 before and after the aggregation of 1 μM Aβ42 with and without 10074-G5 using the W0-2 antibody indicates sequestration of soluble Aβ42. Blotting was performed in triplicate. Fit (**D**) and quantification (**E**) used to estimate the concentration of soluble Aβ42 remaining at the end of the aggregation reaction from (C). The dashed curve in (E) represents fit to eq. S14, describing the equilibrium concentration of unreacted monomer from a competitive binding of free monomers to fibril ends and inhibitor. Using this simple fit, we determined the fitted affinity of 10074-G5 for the soluble material to be *K*_d_ = 7 ± 1 μM. Intrinsic fluorescence profiles of Tyr^10^ of 5 μM Aβ42 in the absence (**F**) and presence (**G**) of 1:1 10074-G5 over 1 hour show that 10074-G5 delays an increase in fluorescence, suggesting that 10074-G5 inhibits early aggregation events including oligomerization and multimerization. Error bars represent ± SDs in (A) and (E).

We measured the kinetics of Aβ42 aggregation at a concentration of 1 μM in the absence and presence of increasing concentrations of 10074-G5. Measurements were performed by means of a fluorescent assay based on the amyloid-specific dye thioflavin T (ThT), which reports on the overall fibril mass formed during the aggregation process (*3*, *35*–*38*). While the range of possible concentrations of Aβ42 and 10074-G5 were restricted for the NMR experiments due to a lack of sensitivity and limited solubilities of both Aβ42 and 10074-G5, the high sensitivity of ThT enabled us to work with lower concentrations of Aβ42 as compared to the NMR experiments and thereby probe higher ligand:protein ratios. We found that 10074-G5 has a notable effect on Aβ42 aggregation (Fig. 4, A and B). Specifically, the data show that the final value of the ThT fluorescence, which corresponds to the end point of the aggregation reaction, is dependent on the concentration of the compound (Fig. 4A). The observation of a significant decrease in the final ThT intensity could be due to several nonmutually exclusive possibilities including (i) interference of the ThT signal by 10074-G5, (ii) formation of soluble off-pathway aggregates, and (iii) sequestration of Aβ42 during the aggregation process (*7*).

Given the fact that 10074-G5 is a colored compound, we sought to investigate whether the decrease in the fluorescence intensity of ThT was exclusively due to an interference of 10074-G5 with the dye or also due to a decrease in the mass of the fibrils formed during the aggregation process. To this end, we performed a ThT-independent dot-blot assay in which we explicitly measured the quantity of soluble Aβ42 over 15 hours in the presence and absence of 10074-G5 using the W0-2 antibody, which binds to Aβ (Fig. 4, C to E). The solubility was determined by measuring the amount of Aβ42 that did not sediment after 1 hour of ultracentrifugation at 100,000 rpm. We observed that in the presence of a 20-fold excess of 10074-G5, approximately 40% of the total amount of Aβ42 remained in a soluble form (Fig. 4, D and E). These experiments indicate that not all Aβ42 monomers are incorporated in ThT-binding fibrils at the end of the aggregation process and, thus, that the presence of 10074-G5 sequesters Aβ42 in its soluble form. These dot-blot data can be explained by an equilibrium model of competitive binding, where monomers can bind both to amyloid fibril ends and to 10074-G5 (Materials and Methods; Fig. 4E). A fit of the dot-blot data to this equilibrium model (eq. S14) yields an affinity of 10074-G5 for the monomers of *K*_d_ = 7 ± 1 μM, a value consistent with that determined independently from the BLI experiments (*K*_d_ = 6 μM), especially considering that in the BLI setup Aβ42 is constrained on a surface (Fig. 1B), whereas in the dot-blot measurement the binding occurs in solution. We further confirmed the observation that Aβ42 remains soluble by exploiting the intrinsic fluorescence of Tyr^10^ in the Aβ42 sequence. By monitoring the aggregation of 5 μM Aβ42 from its monomeric form over 1 hour, the fluorescence intensity of Tyr^10^ increases considerably (Fig. 4F) as it becomes buried in a hydrophobic environment in the aggregated state (*39*). This experiment reports on early aggregation events that may be invisible to ThT, which is specific for cross–β sheet content, as early aggregates such as oligomers or multimers may lack β sheet structure (*40*). We observed, however, that in the presence of an equimolar concentration of 10074-G5, the fluorescence intensity remains constant over time (Fig. 4G), thereby suggesting that Aβ42 does not self-associate in the presence of 10074-G5.

To further demonstrate that 10074-G5 alters the kinetics of aggregation, we performed three-dimensional (3D) morphological analyses of fibrils using high-resolution and phase-controlled (*41*) atomic force microscopy (AFM) on the time scale of the aggregation process (Fig. 4B and fig. S7A). Single-molecule statistical analysis of the aggregates in the 3D maps shows that Aβ42, both in the absence and presence of 10074-G5, forms nonmature aggregates with average cross-sectional diameters of approximately 2 to 3 nm, and mature fibrillar aggregates with average diameters of approximately 5 to 6 nm, as previously observed (*42*, *43*). It has been shown that fibrillar species with diameters less than 6 nm lack a complete (or mature) cross–β sheet structure stabilized by a tight network of intermolecular hydrogen bonding, as compared to mature fibrillar aggregates (*42*). Notably, we observed that at the same time point of aggregation, the fibrillar aggregates formed in the presence of 10074-G5 had smaller cross-sectional diameters than those formed in its absence, with a significantly higher abundance of nonmature species with respect to mature fibrillar species. These data suggest that the process of fibril formation and maturation of cross–β sheet structure in the presence of this compound is considerably slower than in its absence (Fig. 4B and fig. S7A) (*43*).

### 10047-G5 does not chemically modify Aβ42

To determine whether or not the binding of 10074-G5 to Aβ42 is covalent or induces other chemical modifications, we performed mass spectrometry on Aβ42 incubated in the presence and absence of 10074-G5. Samples were incubated overnight at 37°C and then spun down using an ultracentrifuge (Materials and Methods). The supernatant and resuspended pellet of the aggregation reactions were analyzed by matrix assisted laser desorption/ionization (MALDI) mass spectrometry (fig. S7B). No mass increase was observed following the incubation with 10074-G5, indicating that its presence does not result in detectable covalent chemical modifications to Aβ42.

### 10074-G5 inhibits all microscopic steps of Aβ42 aggregation

**Fig. 5:**
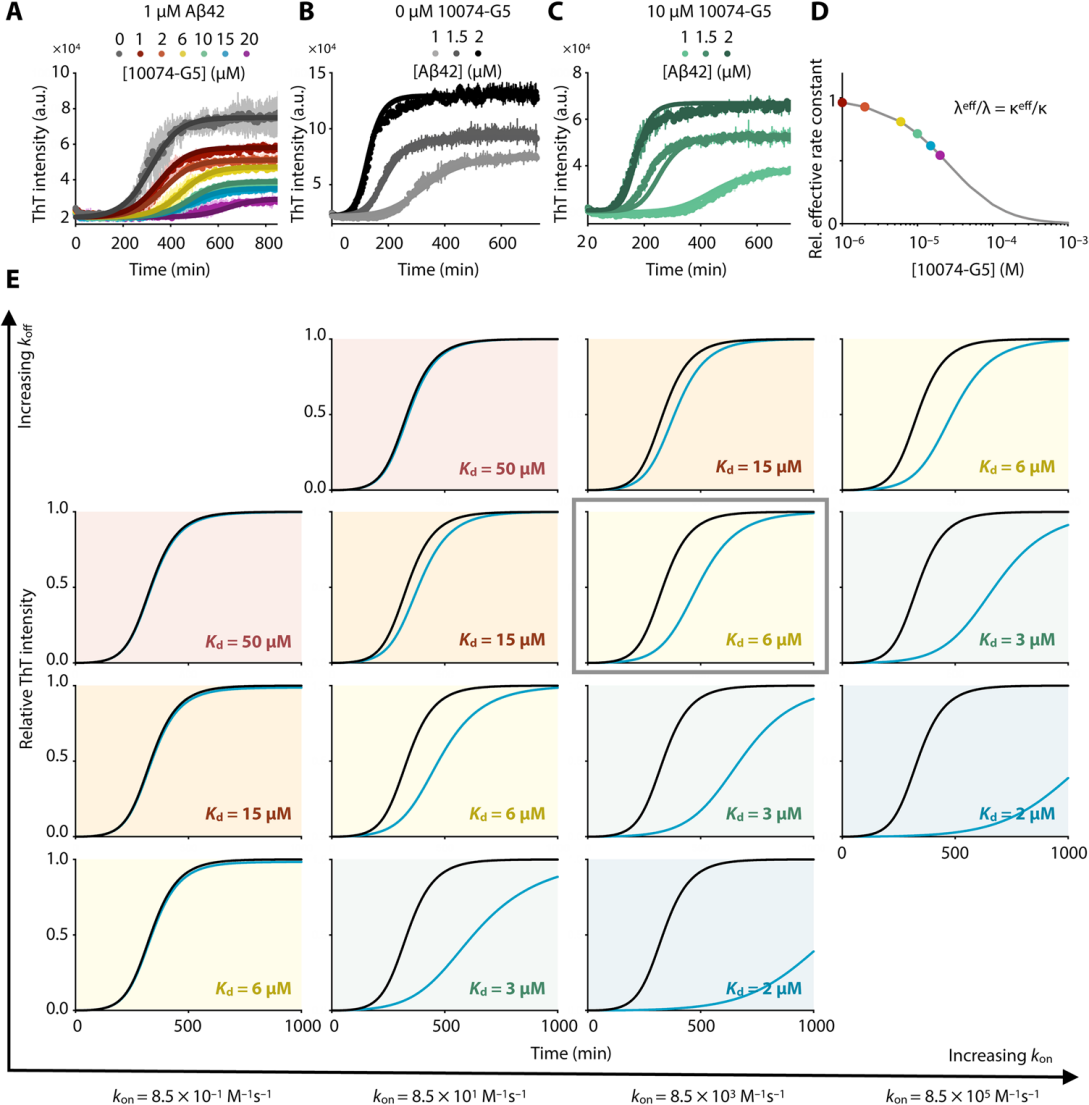
10074-G5 inhibits Aβ42 aggregation primarily by monomer sequestration. (**A**) Global fit of ThT kinetic curves to a monomer sequestration model (eq. S12), in which 10074-G5 affects the aggregation by binding free monomers. Measurements are those shown in Fig. 4A. The theoretical curves are obtained using eq. S11 with unperturbed kinetic obtained from (B) leaving *K*_d_ as the only global fitting parameter (eq. S15). The global fit yields *K*_d_ *=* 40 μM. Global fits to eq. S15 were performed on normalized data to extract changes in the rate parameters in the presence of 10074-G5. (**B**) Global fit to eq. S11 and eq. S12 of ThT kinetic traces of the aggregation reaction for increasing concentrations of Aβ42 (1, 1.5, and 2 μM) in the absence of 10074-G5. Measurements were taken in duplicate or triplicate. (**C**) Overlay of theoretical kinetic curves from (A) with independent ThT kinetic traces of the aggregation reaction for increasing concentrations of Aβ42 (1, 1.5, and 2 μM) in the presence of 10 μM 10074-G5. Solid curves are predictions of the kinetic monomer sequestration model using the same rate parameters and inhibitor binding constant as in (A) and no fitting parameters. Measurements were taken in duplicate or triplicate. (**D**) Effective rates of aggregate proliferation through primary (λ) and secondary (κ) nucleation in the presence of varying concentrations of 10074-G5 determined using the global fit in (A). Error bars represent ± SDs in (A to C). (**E**) Phase diagram illustrating numerical solutions to the kinetic equations for different *k*_on_ and *k*_off_ rates. Curves represent kinetic aggregation traces of 1 μM Aβ42 in the absence (black) and presence of 2 μM compound (blue). Diagonals correspond to constant values of *K*_d_. The values of *k*_+_*k*_n_ and *k*_2_*k*_+_ are the same as those shown in (A).

To better understand the mechanism of inhibition of Aβ42 aggregation by 10074-G5, we performed a kinetic analysis on the ThT aggregation traces. Figure 5A shows the ThT kinetic curves, and from the data, we observe that 10074-G5 slows down the aggregation reaction in a concentration-dependent manner, consistent with the AFM results, confirming a delay in the aggregation process of Aβ42 (Fig. 4B and fig. S7A). Furthermore, our dot-blot analysis shows that a fraction of the total monomer concentration is left unreacted at the end of the aggregation reaction (Fig. 4, C to E). The simplest model that accounts both for the delay of aggregation and for the fact that not all Aβ ends up in the fibrils is one in which monomer is effectively removed from the reaction by the inhibitor. The final load on fibrils depends on the amount of free monomer available for the reaction. Furthermore, reducing the pool of available monomers slows down the rates of all aggregation steps, as monomers are involved in all microscopic steps.

Models of inhibition in which fibril surfaces or fibril ends are targeted by inhibitors would explain the delay in aggregation, but not the decrease fibril load (fig. S3D). By using explicit rate laws (*38*) for describing this inhibition model by monomer sequestration, we sought to understand whether our data could be explained by 10074-G5 exclusively binding the monomer, and in this manner, reducing the concentration of monomers available for the overall aggregation reaction and each microscopic step of aggregation (see the Supplementary Materials). Specifically, we first fitted the measured aggregation kinetics in the absence of 10074-G5 to a kinetic model of Aβ42 aggregation (see the Supplementary Materials and eq. S11) (*38*) to estimate the values of the unperturbed rates for primary nucleation, elongation, and secondary nucleation. We then formulated a master equation model for inhibited aggregation kinetics in the presence of 10074-G5 (eq. S12). We derived explicit integrated rate laws describing inhibited kinetics (eqs. S12 to S15 and fig. S8), which we used to fit the experimental ThT data in the presence of 10074-G5. For this analysis, we implemented the unperturbed rate constants for aggregation, leaving the value of *K*_d_ as the only fitting parameter. We performed a global fit to the normalized data, as described previously (*44*); all ThT profiles at increasing concentrations of 10074-G5 were not fit individually, but rather using the same choice of *K*_d_, with the dependence of the various rate constants on the concentration of 10074-G5 being captured in the integrated rate law through eq. S15. The result of this global fit, shown in Fig. 5A, accounts for the retardation of aggregation in a global manner using one single parameter (*K*_d_) and explains the observed decrease in final fibril load. Furthermore, this fit yields an affinity value of *K*_d_ = 40 μM, consistent with other affinities reported for small-molecule binders of disordered proteins (*22*). It is interesting to note that this estimated affinity is considerably weaker than small-molecule binders of structured proteins, which are often in the nanomolar range. It may seem that such a weak binder of Aβ would have little inhibitory effects. However, we note that the level of inhibition in general depends not on the absolute value of *K*_d_, but on the combined parameter *K*_d_/[*C*], where [*C*] is the drug concentration, provided that the rate of binding (*k*_on_[*C*]) is sufficiently fast as compared to the overall time scale of aggregation, 1/κ (see eq. S11 for a definition of κ) (*45*). Therefore, it is possible to have effective inhibition even for small molecules with *K*_d_ values in the micromolar range, provided that [*C*] is on the same order of magnitude as *K*_d_ (see Fig. 5E).

The analysis of experimental aggregation data in the presence of increasing concentrations of inhibitor using our integrated rate law thus yields an independent method for determining the binding constant of 10074-G5 to the monomers. To provide further support to this analysis, we varied the concentration of monomeric Aβ42 (1, 1.5, and 2 μM) and recorded kinetic traces of aggregation in the absence (Fig. 5B) and presence of 10 μM 10074-G5 (Fig. 5C). Using the rate parameters determined from the uninhibited kinetics and the same value of *K*_d_ obtained from the global fit shown in Fig. 5A, we find that the time course of aggregation predicted by our monomer sequestration model are in good agreement with the independent experimental data (Fig. 5C).

A key prediction from the monomer sequestration model is that a monomer-interacting compound should interfere with all three microscopic steps of aggregation. We find that the presence of an inhibitor that binds monomers quickly compared to the overall aggregation rate does not affect the topology of the reaction network. As a result, the inhibited kinetics can be interpreted in terms of effective rates of aggregation that depend on the concentration of inhibitor (eq. S15). In Fig. 5D, we show the values of the effective rates of aggregate proliferation through primary (λ) and secondary (κ) nucleation pathways as a function of the concentration of 10074-G5 predicted by this model (see eq. S11 for a definition of λ and κ). The monomer sequestration model also predicts that the effective rate of elongation should be reduced, although to a lesser extent than the nucleation pathways, which have a stronger monomer concentration dependence. To test this prediction, we performed seeded aggregation experiments in the presence of preformed Aβ42 fibrils to obtain independent measurements of the effective elongation rate as a function of 10074-G5 concentration. We observed that 10074-G5 indeed decreases the effective rate of fibril elongation (fig. S3B), consistent with the monomer sequestration mechanism. As our understanding of the complex aggregation reaction network of Aβ42 improves, we anticipate that more detailed models capable of describing 10074-G5’s inhibition will become available, including the ability to account for the role of oligomer binding.

We also sought to understand whether 10074-G5 binds monomeric Aβ40 with a comparable affinity to that of Aβ42. To address this question, we applied the monomer sequestration model as used in Fig. 5A to fit the inhibitory effects of 10074-G5 on aggregation of 10 μM Aβ40. From this analysis, we extracted an affinity constant of 10074-G5 for Aβ40 of 10 μM, a similar value to that obtained for Aβ42 (fig. S3A). Given the increased toxicity of Aβ42 as compared to Aβ40, we anticipate the optimization of small molecules more specific for monomeric Aβ42 over Aβ40.

### Characterization of the binding of 10074-G5 to stabilized Aβ40 oligomers

Next, we probed whether 10074-G5 alters the behavior of oligomeric species of Aβ. Although it is extremely challenging to determine whether 10074-G5 modifies the oligomeric intermediates of Aβ42 formed on pathway to aggregation, which are transient, heterogenous species, it is possible to carry out this analysis more readily on oligomers of Aβ40 stabilized using Zn^2+^ (*46*). Thus, we next considered whether or not 10074-G5 can alter the behavior of these stabilized, preformed oligomeric species. We incubated preformed oligomers in the presence of 10074-G5, centrifuged the samples, and measured the quantities of Aβ40 in the pellet and in the supernatant by using SDS–polyacrylamide gel electrophoresis (SDS-PAGE, fig. S9A). The results indicate that these preformed oligomers did not dissociate in the presence of 10074-G5. Furthermore, 10074-G5 was found not to alter the turbidity of solutions in which they were present as measured by their absorbance profiles (fig. S9B), suggesting that 10074-G5 does not cause such species to change detectably in size. Last, dot blots of preformed oligomeric samples in the presence and absence of the compound using the OC-antibody, which binds to β sheets (*47*), show that the oligomers maintain their characteristic conformations (fig. S9C). Because of the colored nature of 10074-G5, it was neither possible to characterize the oligomers in the presence of the compound with dynamic light scattering nor analytical ultracentrifugation measurements. Together, these data suggest that 10074-G5 does not disaggregate the preformed oligomeric species nor cause them to undergo further assembly. Nevertheless, it remains possible that this compound affects the evolution of oligomer populations formed during the aggregation reaction, potentially inhibiting their conversion into fibril-competent species.

### 10074-G5 inhibits Aβ42 aggregation in a *C. elegans* model of Alzheimer’s disease

**Fig. 6:**
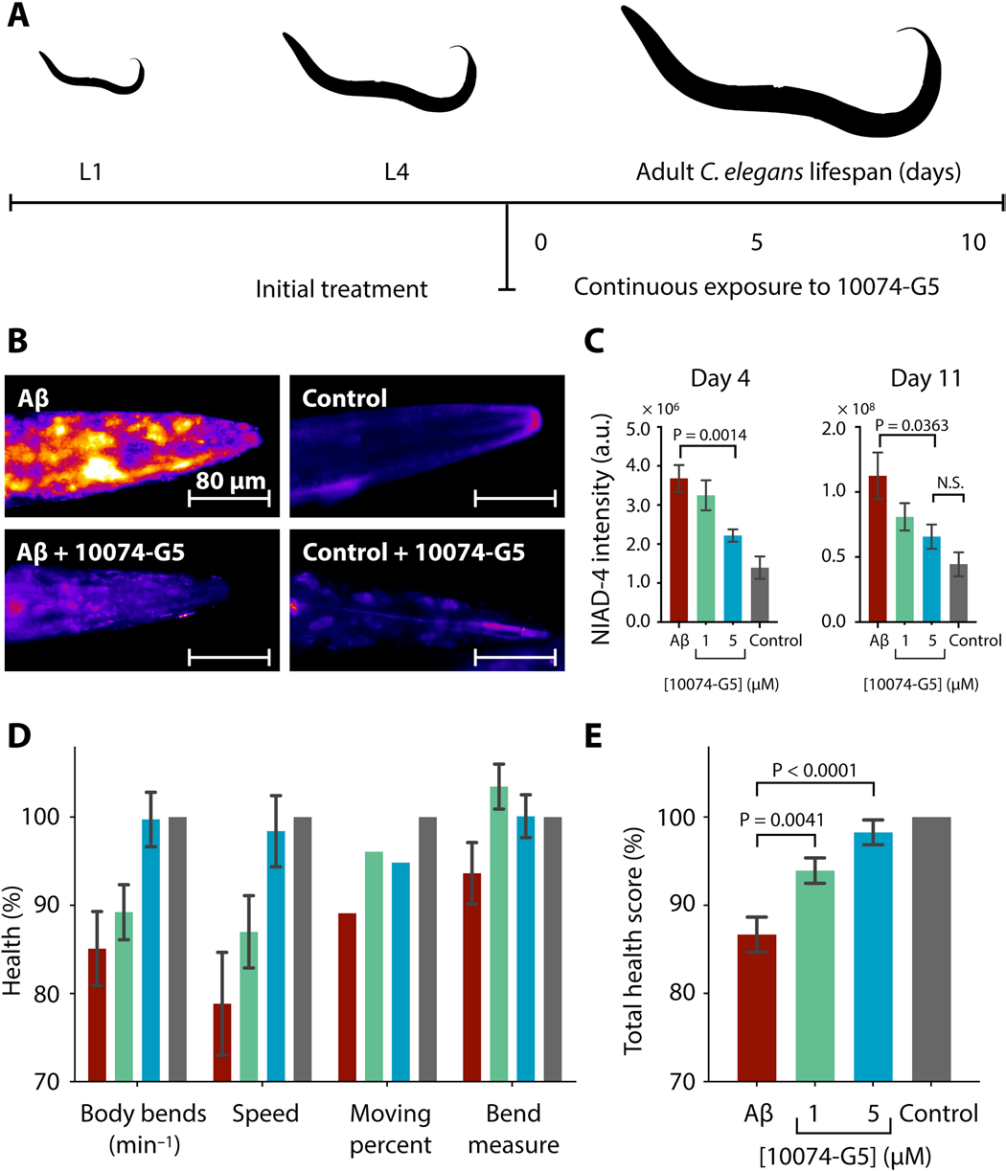
10074-G5 is effective in reducing functional impairment in a *C. elegans* model of Aβ42 toxicity. (**A**) Treatment profile used for the *C. elegans* experiments. (**B**) NIAD-4 staining of *C. elegans* aggregates in the presence and absence of 5 μM 10074-G5. (**C**) Quantification of NIAD-4 intensity shown in (B). *n* ≥ 9. (**D**) Health scores (%) for the rate of body bends, speed of movement, moving percent, and magnitude of body bends at day 6 of adulthood. The colors are the same as those shown in (C). Scores are normalized to the N2 control strain (gray). *n* = 150. (**E**) Combined total health scores [average of health scores in (D)]. In all panels, error bars represent ± SEM. *P* values were determined by two-tailed Student’s *t* test. 10074-G5 shows minimal movement effects on wild-type *C. elegans* (fig. S10).

To determine whether 10074-G5 can inhibit the formation of Aβ42 aggregates in vivo, we tested its effects using a C. elegans model of Aβ42-related toxicity (GMC101; Fig. 6 and fig. S10), in which age-progressive paralysis was induced by overexpression of Aβ42 in the body wall muscle cells (*48*). The N2 wild-type strain (*49*) was used as a control.

10074-G5 was administered to worms from larval stage L4, and then continuously throughout their lifespan (see Materials and Methods and Fig. 6A). First, we quantified the aggregates in the animals by means of an amyloid specific fluorescence probe, NIAD-4 (Fig. 6, B and C). These results show that the administration of 10074-G5 resulted in a lower aggregate load. Then, we monitored a number of phenotypic readouts of worm health, including body bends per minute, the extent of the bending motion, the speed of movement, and also the rate of paralysis (shown as the percent of worms which move). We found that 10074-G5 improved all of these characteristic behavioral parameters in worms expressing Aβ42 in a dose-dependent manner when compared to the untreated worms (Fig. 6, D and E).

To ensure that 10074-G5 does not affect the expression of Aβ42, we performed control experiments using the OW450 strain (*50*), in which the expression of the yellow fluorescent protein (YFP) is controlled by the same gene as the GMC101 strain. We observed that treatment with 10074-G5 does not affect YFP fluorescence in live animals (fig. S10, B and C) nor levels of YFP in *C. elegans* lysates (fig. S10D). Similarly, we also measured levels of Aβ42 in GMC101 *C. elegans* lysates and observed no differences with and without treatment of 10074-G5 (fig. S10E).

Together, these results demonstrate that the administration of 10074-G5 increases the health of this *C. elegans* model of Aβ42-mediated dysfunction and results in the presence of a smaller number of amyloid aggregates. These findings are consistent with the observation of the inhibition of the aggregation of Aβ42 in the presence of 10074-G5 from the in vitro studies (Figs. 4 and 5). Furthermore, these results suggest that the combination of in silico, in vitro, and in vivo drug discovery methods holds promise for the identification of novel small molecules which inhibit the toxic behavior of disease-related disordered proteins.

## DISCUSSION

We have characterized the binding of the small-molecule 10074-G5 to monomeric Aβ42 using a combination of experimental approaches and integrative structural ensemble determination methods. The real-time, dose-dependent responses that we have observed in the BLI experiments demonstrate that 10074-G5 binds Aβ42 in its monomeric form (Fig. 1B and fig. S1). The NMR experiments and the metadynamic metainference simulations have illustrated that this binding is distinct from most small-molecule interactions with structured proteins. In particular, we have observed that 10074-G5 does not bind to a single binding site, but rather binds transiently to many different sites (Figs. 2 and 3, and fig. S6). We have also found 10074-G5 induces Aβ42 to adopt many different conformations, keeping it disordered and more solvent-exposed (Fig. 1, C and D) in the bound form. It appears that this interaction is minimally driven by enthalpy, and thus is largely entropic (fig. S2, B and C) and that some of this entropy arises from the increase in conformational entropy of Aβ42 (Fig. 3) via the entropic expansion mechanism (*18*). This structural insight may open new routes for inhibitors of pathogenic disordered proteins and small-molecule modulators of liquid-liquid phase separation involving disordered regions. In particular, a greater understanding of how one could identify small molecules that alter conformational entropy of disordered proteins without extensive off-target effects holds great therapeutic potential.

In addition, we have characterized the effects of 10074-G5 on amyloid aggregation in vitro using a range of biophysical and kinetic theory techniques. This analysis has revealed that as a result of the interaction of 10074-G5 with monomeric Aβ42, this small molecule also reduces the extent to which monomeric Aβ42 contributes to aggregation, thereby effectively slowing down all microscopic aggregation rates. Our kinetic analysis of aggregation inhibition by monomer sequestration highlights that effective inhibition depends on the interplay of two combined parameters, namely, a thermodynamic and kinetic one: *K*_d_/[*C*] and *k*_on_[*C*]/κ (Fig. 5E) (*45*). Thus, for a small molecule with a given affinity for monomeric Aβ42, increasing the *k*_on_ represents a promising inhibitor optimization strategy. We also anticipate future work to understand the relationship between *k*_on_ and the change in conformational entropy of Aβ42 toward improved binders of monomeric disordered proteins. Last, we show that 10074-G5 is highly effective at reducing the Aβ42 aggregate load and its associated toxicity in a *C. elegans* model of Alzheimer’s disease (Fig. 6).

The results that we have reported indicate the importance of developing a detailed understanding of the mechanisms of interaction between disordered proteins and small molecules. Increasing our knowledge about these mechanisms could lead, in turn, to the development of new therapeutic approaches for the many diseases in which disordered proteins are involved.

## MATERIALS AND METHODS

### BLI experiments

A super streptavidin biosensor (FortéBio, Menlo Park, USA) was coated with monomeric N-terminally biotinylated Aβ42 (15 μg/ml; AnaSpec, Fremont, USA) by overnight incubation a solution at 5°C. Control biosensors were incubated with the same concentration of biocytin. The tips were then rinsed by incubation in buffer for 3 hours at room temperature. The binding and dissociation between immobilized Aβ42 and various concentrations of 10074-G5 was monitored for 200 and 500 s, respectively, at 37°C using an Octet Red96 (ForteBio, Menlo Park, USA). This process was repeated six times (fig. S1, A and B). The binding of buffer to a Aβ42-functionalized biosensor was subtracted to account for baseline drift. Data were analyzed using GraphPad Prism 8. Dissociation data were first globally fit using a one-phase exponential decay to determine a preliminary *k*_off_ value. This value was then used as an initial input value to determine the global *k*_on_ and *k*_off_ rates.

### 2D H^N–BEST^CON NMR experiments

^13^C, ^15^N uniformly labeled, recombinant Aβ42 peptide (the 42-residue variant lacking the N-terminal M; see the “Preparation of recombinant Aβ peptides” section) was purchased from rPeptide and prepared following the manufacturer’s instructions. Twenty micromolar samples were prepared in phosphate-buffered saline (PBS) (pH 7.50) and 1% dimethyl sulfoxide (DMSO), with 5% D_2_O (Sigma-Aldrich) for the lock. 2D H^N–BEST^CON measurements (*25*) were performed at 16.4 T on a Bruker Avance spectrometer operating at 700.06 MHz ^1^H, 176.03 MHz ^13^C, and 70.9 MHz ^15^N frequencies, equipped with a triple-resonance cryogenically cooled probehead optimized for ^13^C-direct detection (at the Centro di Risonanze Magnetiche, Florence, Italy). Each 2D H^N–BEST^CON spectrum was acquired with 64 scans. The dimensions of the acquired data were 1024 (^13^C) × 116 (^15^N) points. The spectral width was 29.9 × 33.9 ppm for F_2_ and F_1_, respectively. The relaxation delay was set to 0.3 s. 2D H^N–BEST^CON measurements were repeated with the same parameters except for the inclusion of a weak presaturation of the solvent signal during the relaxation delay. Under these conditions, signals of amide nitrogen whose directly bound protons are in fast exchange with the solvent are attenuated. This approach was tested on a well-characterized protein (ubiquitin) and then used for the study of the Aβ42 peptide with and without the addition of 10074-G5. 1D ^1^H and 2D BEST TROSY (*51*) spectra were acquired before and after measurements were taken to ensure that minimal aggregation had occurred during the course of the measurement. Experimental data were acquired at 5° and 15°C using Bruker TopSpin 3.1 software and processed with Sparky 3.115.

### Metadynamic metainference simulations

To generate the structural ensembles, we used an integrative approach that incorporates NMR chemical shift data into molecular dynamics simulations. To this end, we used metadynamic metainference, which compensates for the inaccuracies of the force field, accounts for errors in experimental data, and enhances sampling (*28*, *29*). All-atom metadynamic metainference simulations (*28*) of the unbound and bound form of Aβ42 were performed using GROMACS 2018.3 (*52*) patched with PLUMED library 2.6.0-dev (git: 0edcfb268569) (*53*), the CHARMM22* force field (*54*), and TIP3P water model (*55*). The initial conformation of Aβ42 was prepared as a linear peptide using PyMOL (https://pymol.org/2/). A preliminary in vacuo molecular dynamics simulation was performed for 1 ns to collapse the extended conformation. This structure was solvated in a rhombic dodecahedron box with an initial volume of 362 nm^3^ containing 11,746 water molecules. The solvated system was minimized using the steepest descent algorithm with a target maximum force of 1000 kJ mol^−1^ nm^−1^. A pool of 48 initial conformations was extracted from a preliminary 2-ns simulation at 600 K in the NVT ensemble. Equilibration was then performed in the canonical (NVT) ensemble for 500 ps at 278 K using the Bussi-Donadio-Parrinello thermostat (*56*) and for 500 ps at 278 K in the isothermal-isobaric (NPT) ensemble using Berendsen pressure coupling (*57*) with position restraints on heavy atoms. Production runs were executed in the NPT ensemble at 278 K using the Parrinello-Rahman barostat (*58*). A time step of 2 fs was used together with LINCS constraints on all bonds (*59*). The van der Waals interactions were cut off at 1.2 nm, and the particle-mesh Ewald method was used for electrostatic interactions (*60*). Bound simulations were performed as described above, using the starting structures obtained from the NVT equilibration at 600 K. The 10074-G5 molecule was added to a corner of the box and the system resolvated with 11,734 water molecules. The system was then minimized and equilibrated using the procedures described above. Preliminary parameters for 10074-G5 were taken from the CGenFF software (*61*), and those with any penalty were explicitly reparameterized using the Force Field Toolkit (*62*) and Gaussian 09 (www.gaussian.com) (see the Supplementary Materials and fig. S4).

Chemical shifts were back calculated at each time step using CamShift (fig. S5) (*63*). Given that the error of the CamShift predictor is greater than the chemical shift perturbations upon addition of the compound, the same chemical shifts were used to restrain both the unbound and bound simulations. A Gaussian noise model with one error parameter per nucleus type was used in the metainference setup, along with an uninformative Jeffreys prior for each error parameter (see the Supplementary Materials) (*28*). The metainference ensembles for the unbound and bound simulations were simulated using 48 replicas each.

Parallel bias metadynamics (*64*) with the well-tempered (*65*) and multiple-walker (*66*) protocols were performed using a Gaussian deposition stride of 1 ps, an initial height of 1.2 kJ/mol, and bias factors of 24 and 49 for the unbound and bound simulations, respectively. In the unbound simulations, we used six collective variables (CVs) to enhance the conformational sampling of Aβ42 (see the Supplementary Materials). In the bound simulations, we also included 14 CVs to enhance the conformational sampling of contacts between the compound and the peptide and four CVs to enhance sampling of soft dihedrals in the small molecule (see the Supplementary Materials). Unbound and bound simulations were run for an accumulated time of 27.8 and 28.2 μs, respectively, until convergence was reached (see the Supplementary Materials and Fig. 3C). For details on the analysis, see the Supplementary Materials.

### Preparation of recombinant Aβ peptides

Recombinant Aβ(M1-42) (MDAEFRHDSGY EVHHQKLVFF AEDVGSNKGA IIGLMVGGVVIA) and Aβ(M1-40) (MDAEFRHDSGY EVHHQKLVFF AEDVGSNKGA IIGLMVGGVV), here, referred to as Aβ42 and Aβ40, respectively, were prepared by expression in *Escherichia coli* BL21 (DE3) Gold Strain (Agilent Technologies, Santa Clara, USA) (*37*). The resulting inclusion bodies were dissolved in 8 M urea, ion exchanged in batch mode on diethylaminoethyl cellulose resin, lyophilized, and then further purified with a Superdex 75 HR 26/60 column (GE Healthcare, Chicago, USA). Fractions containing the recombinant protein, as determined by SDS-PAGE, were combined and lyophilized again. To ensure we were working with highly purified monomeric species containing extremely low quantities of aggregated forms of the peptides, size exclusion chromatography was carried out directly before the experiments were performed. Aβ40 and Aβ42 solutions were prepared by dissolving the lyophilized peptide in 6 M GuHCl and incubating on ice for 3 hours. The solutions were then purified using a Superdex 75 Increase 10/300 GL column (GE Healthcare, Chicago, USA) at a flow rate of 0.5 ml/min and eluted in 20 mM sodium phosphate buffer (pH 8) supplemented with 200 μM EDTA. The center of the peak was collected, and the concentrations of the peptides were determined from the integration of the absorbance peak using ε_280_ = 1495 L mol^−1^ cm^−1^.

### Preparation of small molecules

10074-G5 was obtained from Sigma-Aldrich (St. Louis, MO, USA). The molecules were dissolved in 100% DMSO and then diluted in solutions of Aβ40 or Aβ42 to reach a maximum final DMSO concentration of 1.5%. The total DMSO concentration was matched in the control solutions in all experiments.

### ThT aggregation kinetics

Monomeric Aβ40 or Aβ42 were diluted with buffer and 20 μM ThT from a 2 mM stock and increasing amounts of 10074-G5. Samples were prepared using LoBind Eppendorf tubes (Sigma-Aldrich, MO, St. Louis, USA) on ice. Fibrils for seeding experiments were prepared by incubating monomeric Aβ42 at 37°C overnight. The concentration of fibrils (in monomer equivalents) was assumed to be the initial concentration of monomer. These preformed fibrils were added to a freshly prepared monomer solution to give a final concentration of 15% fibrils.

Samples with or without seed fibrils were pipetted into multiple wells of a 96-well half-area, low-binding polyethylene glycol coating plate (Corning 3881, Sigma-Aldrich) with a clear bottom, at 90 μl per well. Plates were sealed with aluminum sealing tape (Corning, Sigma-Aldrich) to prevent evaporation and then placed at 37°C under quiescent conditions in a plate reader (CLARIOstar; BMG Labtech, Ortenberg, Germany). ThT fluorescence was measured through the bottom of the plate using 440- and 480-nm excitation and emission filters, respectively. ThT fluorescence was followed with multiple replicates as specified in the corresponding figure captions. For analysis of ThT kinetics see the Supplementary Materials.

### Mass spectrometry

Monomeric Aβ42 was diluted in the aggregation buffer (described above) to a concentration of 15 μM in the presence and absence of 30 μM 10074-G5. Samples were incubated overnight at 37°C under quiescent conditions to mimic the aggregation experiments. The samples were then spun down using an ultracentrifuge at 100,000 rpm for 1 hour at 25°C to separate the supernatant and pellet. GuHCl (6 M) was used to dilute the supernatant by 50% and resuspend the pellet. Samples were analyzed by MALDI mass spectrometry at the Protein and Nucleic Acid Chemistry Facility at the Department of Biochemistry, University of Cambridge.

### Dot-blot assay

Blotting was performed using the Aβ42 sequence-specific antibody (W0-2, MABN10, Millipore, Burlington, USA). Samples were removed from a solution containing 2 μM Aβ42 in the presence and absence of 3- and 10-fold equivalence of 10074-G5. To ensure only the monomer was placed on the blots, samples were spun down using an ultracentrifuge at 100,000 rpm for 1 hour at 25°C using a TLA100 rotor. Two microliters of the supernatant was pipetted onto a nitrocellulose membrane (0.2 μM; Whatman). After drying, the membrane was blocked with 5% (w/v) bovine serum albumin (BSA) in PBS [8 mM Na_2_HPO_4_, 15 mM KH_2_PO_4_, 137 mM NaCl, 3 mM KCl (pH 7.4), and PBS] overnight at 5°C, followed by three 15-min washes with PBS at room temperature. The membrane was then immunized with a 1:1000 dilution of WO-2 anti-Aβ antibody in PBS with 5% BSA overnight at 5°C, followed by three 15-min washes with PBS at room temperature. The membrane was then incubated for 2 hours at room temperature in PBS supplemented with 0.05% Tween 20 and an anti-mouse–Alexa Fluor 594 secondary antibody conjugate (Thermo Fisher Scientific, Waltham, USA) at room temperature and then washed three times with PBS supplemented with 0.05% Tween 20. Fluorescence detection was performed using Typhoon Trio Imager (GE Healthcare, Chicago, IL, USA). Blots were quantified using ImageJ. Data were fit to a competitive binding equilibrium model between free monomers and fibrils (eq. S14). In this model, monomers are either free, aggregated (i.e., part of a fibril), or bound to 10074-G5; the binding of the compound to the monomer is described by a single-binding free energy. The binding of monomers to fibril ends is stronger compared to the binding of monomers to the inhibitor. The concentration of free monomer in equilibrium with amyloid fibrils (critical concentration) measured in our experiments was *m*_critical_ = 93 nM, consistent with other reports (*67*). The equilibrium concentration of unreacted soluble monomer after ultracentrifugation measured at varying inhibitor concentrations was fit to eq. S14 with *K*_d_ as a fitting parameter. This procedure yields *K*_d_ = 7 ± 1 μM, as shown in Fig. 4E.

### Atomic force microscopy

Solutions of 1 μM Aβ42 in the presence and absence of 6 μM 10074-G5 were deposited on mica positively functionalized with (3-aminopropyl) triethoxysilane (APTES; Sigma-Aldrich, St. Louis, USA) in the absence of ThT. The incubation times were selected on the basis of the results of the chemical kinetics experiments. The mica substrate was positively functionalized by incubation with a 10-μl drop of 0.05% (v/v) APTES in Milli-Q water for 1 min at ambient temperature, rinsed with Milli-Q water, and then dried by the passage of a gentle flow of gaseous nitrogen (*42*). AFM sample preparation was carried out at room temperature by deposition of a 10-μl drop of protein solution deposited for 2 min to a surface treated with APTES. The samples were rinsed with Milli-Q water, dried with nitrogen gas, and stored in a sealed container until imaging. AFM maps were acquired by means of a NX10 (Park Systems, Suwon, Korea) and a nanowizard2 (JPK Instruments, Berlin, Germany) system operating in tapping mode and equipped with a silicon tip (PPP-NCHR and μmasch) with a nominal radius of 10 nm. Image flattening and single aggregate statistical analysis were performed by SPIP 6 (Image Metrology, Hørsholm, Denmark) software.

### Isothermal titration calorimetry experiments

Measurements were performed using an MicroCal Auto-ITC 200 (GE Healthcare, Chicago, USA) at 15°C. Because of the poor solubility of 10074-G5, monomeric Aβ40 (200 μM) was injected 10 times into a solution containing 7 μM 10074-G5. All solutions were prepared in phosphate buffer (described above) and contained a minimal amount of DMSO (0.2%) to ensure that the compound was soluble. Each injection was 3.5 μl in volume and was made on 3-min intervals. Heats of dilution, obtained by separately injecting the peptide into buffer and buffer into the solution containing 10074-G5, were subtracted from the final data. The corrected heats were divided by the number of moles injected and analyzed using Origin 7.0 software (OriginLab, Northampton, USA).

### Characterization of the interaction of 10074-G5 with stabilized oligomers

Stabilized oligomers were formed from Aβ40 as previously described (*46*). Briefly, 1 mg of lyophilized Aβ40 was dissolved in 300 μl of hexafluoroisopropanol and incubated overnight at 4°C. After solvent evaporation under nitrogen gas, Aβ40 was resuspended in DMSO to a concentration of 2.2 mM and sonicated twice for 10 min at room temperature. The protein sample was diluted to a final concentration of 100 μM in 20 mM sodium phosphate buffer with 200 μM ZnCl_2_ at pH 6.9. After incubation for 20 hours at 20°C, the solution was centrifuged for 15 min at 15,000*g* at room temperature. The pellet containing the oligomers was resuspended in 20 mM phosphate buffer at pH 6.9, with 200 μM ZnCl_2_.

Samples containing 20 and 10 μM preformed Zn^2+^-stabilized Aβ40 oligomers were incubated in the presence and absence of 20 μM 10074-G5 for 1 hour. The turbidimetries of the samples were analyzed using a plate reader (BMG Labtech, Aylesbury, UK) at 600 nm. Measurements were background subtracted against buffer alone in the absence and presence of compound. The protein content within samples was quantified using the sequence-specific WO-2 antibody (see the “Dot-blot assay” section). Similarly, the conformations of the oligomers in the presence and the absence of the compound was probed using the conformation-specific OC antibody (*47*) (AB2286, Millipore, Burlington, USA) using the protocols described above (see the “Dot-blot assay” section).

To determine whether the oligomers had dissociated after the incubation in the presence of the compound, the samples were centrifuged for 15 min at 15,000*g*. The pellet was resuspended in 15 μl of 20 mM phosphate buffer at pH 6.9 with 200 μM ZnCl_2_ and analyzed along with the supernatant by SDS-PAGE.

### *C. elegans* experiments

The following *C. elegans* strains were used: the temperature-sensitive human Aβ-expressing strain dvIs100 [unc-54p:: A-beta-1–42::unc-54 3′-UTR + mtl-2p::GFP] (GMC101), where mtl-2p::GFP causes intestinal GFP expression, and unc-54p::Aβ1–42, which expresses the human full-length Aβ42 peptide in the muscle cells of the body wall. Raising the temperature above 20°C at the L4 or adult stage causes paralysis due to Aβ42 aggregation in body wall muscle (*48*). The N2 wild-type strain was used as a control (*48*, *49*).

Standard conditions were used for the propagation of *C. elegans* (*48*); the animals were synchronized by hypochlorite bleaching, hatched overnight in M9 [KH_2_PO_4_ (3 g/liter), Na_2_HPO_4_ (6 g/liter), NaCl (5 g/liter), and 1 μM MgSO_4_] buffer, and subsequently cultured at 20°C on nematode growth medium (NGM) [1 mM CaCl_2_, 1 mM MgSO_4_, cholesterol (5 μg/ml), 250 μM KH_2_PO_4_ (pH 6), agar (17 g/liter), NaCl (3 g/liter), and casein (7.5 g/liter)] plates seeded with the *E. coli* strain OP50. Saturated cultures of OP50 were grown by inoculating 50 ml of LB (Luria Broth) medium [tryptone (10 g/liter), NaCl (10 g/liter), and yeast extract (5 g/liter)] with OP50 and incubating the culture for 16 hours at 37°C. NGM plates were seeded with bacteria by adding 350 μl of saturated OP50 to each plate and leaving the plates at 20°C for 2 to 3 days. On day 3 after synchronization, the animals were placed on NGM plates containing 5-fluoro-2’deoxy-uridine (FUDR) (75 μM, unless stated otherwise) to inhibit the growth of offspring.

NGM plates containing FUDR (75 μM) were seeded with 350 μl of OP50 culture and grown overnight. After incubating for up to 3 days at room temperature, 2.2-ml aliquots of 10074-G5 dissolved in 1% DMSO at different concentrations were spotted atop the NGM plates. The plates were then placed in a sterile laminar flow hood at room temperature to dry. For the final experiments, worms were transferred onto the 10074-G5–seeded plates directly at larval stage L4, and they were exposed to 10075-G5 for the whole duration of the experiment.

To ensure that the presence of 10074-G5 did not affect the OP50 *E. coli* consumed by the *C. elegans*, we performed a growth assay of the *E. coli* directly from the NGM plates in the presence of 10074-G5 or DMSO after 1 day of incubation at 24°C (fig. S10A). *E. coli* from the NGM plates were added to 4 ml of LB media and diluted to an optical density of 0.25. Then, 3 ml of this starter culture was added to 40 ml of sterile LB media, which was incubated at 37°C and shaking at 180 rpm. Optical density measurements were collected every 30 min, and the experiment was performed in duplicate.

All *C. elegans* populations were cultured at 20°C and developmentally synchronized from a 4-hour egg lay. At 64 to 72 hours after egg lay (time zero), individuals were transferred to FUDR plates and cultured at 24°C to stimulate aggregation, and body movements were assessed over the times indicated. At different ages, the animals were washed off the agar plates with M9 buffer and spread over an OP50 unseeded 9-cm plate. The swimming worms were visualized by using a high-performance imaging lens and a machine vision camera, after which their movements were recorded at a high number of frames per second (fps) for 30 s or 1 min (*68*, *69*). Body bends were then quantified using a tracking algorithm (*69*, *70*). Briefly, after an initial background subtraction, a second (nonadaptive) thresholding procedure was performed, and worms were identified and labeled. The eccentricity, a measure of the ratio of the minor and major ellipse axes, of each tracked worm was then used to estimate the worm bending as a function of time (*69*, *70*). The total health was calculated by averaging the mobility, speed, bend measure, and viability of the worms (*69*, *70*). Total health values were normalized using the values of the control worms. At least 150 animals were examined per condition, unless stated otherwise. All experiments were carried out in triplicate, and the data from one representative experiment are shown in Fig. 6. Control experiments to test the effects of 10074-G5 on the movement of N2 wild-type *C. elegans* are shown in fig. S10 (F and G). Two-tailed Student’s *t* tests (unpaired) were used to calculate *P* values. Statistical analysis was performed using the GraphPad Prism 6 software.

To ensure 10074-G5 did not alter the function of unc-54p, we performed control experiments using the rmIs126 [P(unc-54)Q0::YFP]V (OW450) strain (*50*). In this strain, the YFP is expressed and remains diffusely localized throughout aging. OW450 *C. elegans* were treated in the presence and absence of 5 μM 10074-G5. Live transgenic animals were imaged using Cell Culture plates (Nunc MicroWell 96-Well, catalog no. 165305, Thermo Fisher Scientific) and immobilized using 40 mM NaN_3_ as anesthetic. Images were captured with an EVOS M7000 fluorescence microscope (Thermo Fisher Scientific) with a 10× objective and a YFP filter. Over 100 worms were analyzed per condition. One representative image per condition is shown (fig. S10B). The average fluorescence intensity per worm (fig. S10C) was calculated using ImageJ software (National Institutes of Health). In addition, more than 1000 OW450 animals per condition were frozen in S basal in liquid N_2_, thawed, and resuspended in 500 μl of PBS supplemented with one cOmplete Mini EDTA-free Protease Inhibitor Cocktail tablet (Roche). Animals were then sonicated five times for 45 s (50% cycles at 50% maximum power) on ice with a Bandelin Sonopuls HD 2070 sonicator, followed by 15 min of centrifugation at maximal speed using a bench centrifuge. The supernatant (2.5 μl) was spotted onto nitrocellulose membranes in triplicate and probed using a 1:1000 dilution of the anti-YFP primary antibody (Bio-Rad) using the protocols described above (see the “Dot-blot assay” section). To ensure standard protein loading, parallel blots were probed using an anti–α-tubulin antibody (Abcam) (fig. S10D). Similarly, the amount of Aβ in treated and untreated GMC101 *C. elegans* were quantified as described above using a 1:1000 dilution of the Aβ sequence-specific WO-2 antibody (see the “Dot-blot assay” section and fig. S10E). Approximately 5000 worms were used per condition.

To stain the aggregates within the *C. elegans*, live transgenic animals were incubated with 1 μM NIAD-4 (0.1% DMSO in M9 buffer) for 4 hours at room temperature (*9*). After staining, animals were allowed to recover on NGM plates for about 24 hours to allow destaining via normal metabolism. Stained animals were mounted on 2% agarose pads containing 40 mM NaN_3_ as an anesthetic on glass microscope slides for imaging. Images were captured with a Zeiss Axio Observer D1 fluorescence microscope (Carl Zeiss Microscopy GmbH, Jena, Germany) with a 20× objective and a 49004 ET-CY3/TRITC filter (Chroma Technology Corp, Bellows Falls, USA). Fluorescence intensity was calculated using ImageJ software (National Institutes of Health) and then normalized as the corrected total fluorescence (*9*, *71*). Only the head region was considered because of the high background signal in the intestinal regions. At least 9 animals were examined per condition.

## Supplementary Material

http://advances.sciencemag.org/cgi/content/full/6/45/eabb5924/DC1

Adobe PDF - abb5924_SM.pdf

Small-molecule sequestration of amyloid-β as a drug discovery strategy for Alzheimer’s disease

## SUPPLEMENTARY MATERIALS

Supplementary material for this article is available at http://advances.sciencemag.org/cgi/content/full/6/45/eabb5924/DC1

## Appendix

View/request a protocol for this paper from *Bio-protocol*.
