# Supplementary material for: Small-molecule sequestration of amyloid-β as a drug discovery strategy for Alzheimer’s disease
Source: Sci Adv. 2020 Nov 4;6(45):eabb5924. doi: 10.1126/sciadv.abb5924 (PMC7673680; doi:10.1126/sciadv.abb5924)
Supplement: http://advances.sciencemag.org/cgi/content/full/6/45/eabb5924/DC1 [file supp_6_45_eabb5924__index.html]

Science Advances | Science AdvancesAAASSearchScience AdvancesMenu

## Supplementary Materials

# Small-molecule sequestration of amyloid-β as a drug discovery strategy for Alzheimer’s disease

Gabriella T. Heller, Francesco A. Aprile, Thomas C. T. Michaels, Ryan Limbocker, Michele Perni, Francesco Simone Ruggeri, Benedetta Mannini, Thomas Löhr, Massimiliano Bonomi, Carlo Camilloni, Alfonso De Simone, Isabella C. Felli, Roberta Pierattelli, Tuomas P. J. Knowles, Christopher M. Dobson, Michele Vendruscolo

Download Supplement

**This PDF file includes:**

- Details of 10074-G5 parameterization
- Details of the metainference approach
- Details of the metadynamics setup
- Details of structural ensemble analysis
- Kinetic analysis of experimental aggregation data using a monomer sequestration model
- Figs. S1 to S10
- References

**Files in this Data Supplement:**

- Adobe PDF - abb5924\_SM.pdf
