# Supplementary material for: Small-molecule sequestration of amyloid-β as a drug discovery strategy for Alzheimer’s disease
Source: Sci Adv. 2020 Nov 4;6(45):eabb5924. doi: 10.1126/sciadv.abb5924 (PMC7673680; doi:10.1126/sciadv.abb5924)
Supplement: http://advances.sciencemag.org/cgi/content/full/6/45/eabb5924/DC1 [file supp_6_45_eabb5924__1.pdf]

Science Advances
AAAS

advances.sciencemag.org/cgi/content/full/6/45/eabb5924/DC1

Supplementary Materials for

Small-molecule sequestration of amyloid-β as a drug discovery strategy for Alzheimer's disease

Gabriella T. Heller, Francesco A. Aprile, Thomas C. T. Michaels, Ryan Limbocker, Michele Perni, Francesco Simone Ruggeri, Benedetta Mannini, Thomas Löhr, Massimiliano Bonomi, Carlo Camilloni, Alfonso De Simone, Isabella C. Felli, Roberta Pierattelli, Tuomas P. J. Knowles, Christopher M. Dobson, Michele Vendruscolo*

*Corresponding author. Email: mv245@cam.ac.uk

Published 4 November 2020, *Sci. Adv.* **6**, eabb5924 (2020)
DOI: 10.1126/sciadv.abb5924

This PDF file includes:

Details of 10074-G5 parameterization
Details of the metainference approach
Details of the metadynamics setup
Details of structural ensemble analysis
Kinetic analysis of experimental aggregation data using a monomer sequestration model
Figs. S1 to S10
References

Details of 10074-G5 parameterization. Initial parameters for 10074-G5 compatible with the CHARMM22* force field were determined using the CHARMM General Force Field (CGenFF) (*61*). Parameters with penalties were re-parametrized using the Force Field Toolkit (*62*) and Gaussian 09 (http://www.gaussian.com) as follows: Geometry optimization was performed at the quantum mechanical (MP2/6-31G*) level. Partial atomic charges were derived to reproduce interaction energies and distances with TIP3P water molecules, in a manner consistent with the CHARMM force field. Briefly, to obtain target data, water molecules were positioned either as a hydrogen bond donor or acceptor as previously described (*62*) and their optimal distances and orientations with 10074-G5 were calculated at the HF/6-31G* level of theory. Then, the partial atomic charges of 10074-G5 were optimized so that the molecular mechanics interaction energy and distances match the quantum mechanical data. Target data in form of the Hessian matrix for bonds and angles were obtained at the MP2/6-31G* level of theory and were used to fit the corresponding bonded molecular mechanics parameters. The dihedral target data were generated by performing relaxed scans of all dihedral angles with CGenFF penalties using 15° intervals at the MP2/6-31G* level of theory. Given the coupled nature of dihedrals in this multi-ring system, dihedral parameters were simultaneously fit to this torsional potential energy surface as previously described (*62*). Dihedral fits to target data are shown in fig. S4.

Details of the metainference approach. Metainference is a Bayesian integrative approach that enables incorporating experimental data into molecular dynamics simulations (*29*). The relative weight of experimental data with respect to the molecular dynamics force field is determined by the accuracy, or level of error, in the data. To account for random and systematic errors in the chemical shift data and in the CamShift (*63*) predictor, we used a Gaussian model of noise

$$p\left(d_{n,m}\middle|\{X\},\sigma_{\mathrm{m}}\right)=\frac{1}{\sqrt{2\pi}\sigma_{\mathrm{m}}}\exp\left[-\frac{\left(d_{n,m}-\bar{d}_{n,m}(\{X\})\right)^2}{2\sigma_{\mathrm{m}}^2}\right] \quad \text{(S1)}$$

where $d_{n,m}$ is the n-th data point of the m-th dataset, $\{X\}$ is the set of the conformations of all replicas, $\bar{d}_{n,m}(\{X\})$ is the prediction averaged over our metainference ensemble of 48 replicas, and σ_{m} is an uncertainty parameter. Each experimental dataset contains the chemical shifts of a different nucleus. For both the unbound and bound simulations we used 6 datasets for a total of 230 data points (40 ^{13}Cα chemical shifts, 34 ^{13}Cβ chemical shifts, 40 ^{13}C'chemical shifts, 41 ^{1}Hα chemical shifts, 41^{1}HN chemical shifts, and 34 ^{15}N chemical shifts).

The uncertainty parameter

$$\sigma_{\mathrm{m}} = \sqrt{(\sigma_{\mathrm{m}}^{\mathrm{B}})^2 + \left(\sigma_{\mathrm{m}}^{\mathrm{SEM}}\right)^2} \tag{S2}$$

quantifies the deviation between predicted and measured data and accounts for random and systematic errors as well as errors in the predictor ($\sigma_{\mathrm{m}}^{\mathrm{B}}$), and the statistical error in calculating ensemble averages over a finite number of replicas ($\sigma_{\mathrm{m}}^{\mathrm{SEM}}$). $\sigma_{\mathrm{m}}^{\mathrm{SEM}}$ was initialized to 0.5 and updated with a windowed average calculation of 500 steps (*72*). $\sigma_{\mathrm{m}}^{\mathrm{B}}$ was set to an initial value of 9.0 and sampled using a Monte Carlo algorithm in the range from 0.00001 to 10.0, with a maximal trial move equal to 0.1. For each uncertainty parameter, we used an uninformative Jeffreys prior: $p(\sigma_{\mathrm{m}}) = 1/\sigma_{\mathrm{m}}$.

Details of the metadynamics setup. Parallel bias metadynamics (*64*) was used to enhance sampling of the conformational landscape of Aβ42, in combination with the multiple-walkers protocol (*66*). The collective variables (CVs) used were:

1) The total α-helical content as quantified using the ALPHARMSD keyword in PLUMED. This CV is computed by first generating the set of all possible regions of six consecutive residues within the system and then calculating the root-mean-square distance ($\mathrm{RMSD_i}$) between each segment and an idealized α-helical structure. The CV is then calculated using the following switching function to make it differentiable

$$s_{\alpha\mathrm{Hel}} = \sum_i \frac{1-\left(\frac{\mathrm{RMSD}_i}{\mathrm{RMSD}_0}\right)^8}{1-\left(\frac{\mathrm{RMSD}_i}{\mathrm{RMSD}_0}\right)^{12}} \tag{S3}$$

where the sum runs over all possible α-helical segments and $\mathrm{RMSD}_0 = 0.08$ nm. A Gaussian width equal to 0.64 was used for this CV.

2) The sum of the total parallel and anti-parallel β-sheet content. Total parallel β-sheet content is calculated using the PARABETARMSD keyword in PLUMED. This value is computed by first generating the set of all possible six residue groupings within the system that can form a parallel β-sheet. Within a given protein chain, two segments containing three continuous residues can form a parallel β-sheet if they are separated by a minimum of 3 residues to accommodate a turn. Then, the RMSD_i between each grouping and an idealized parallel β-sheet is calculated. The total parallel β-sheet content is then calculated using eq. S3 above,

where the sum runs over all potential parallel β-sheets. Similarly, the total anti-parallel β-sheet content is calculated using the ANTIBETARMSD keyword in PLUMED. This value is computed by first generating the set of all possible six residue groupings within the system that can form an anti-parallel β-sheet. Within a given protein chain, two segments containing three consecutive residues can form an anti-parallel β-sheet if they are separated by a minimum of 2 residues accommodate a turn. Then, the RMSD_i between each grouping and an idealized anti-parallel β-sheet is calculated. The total anti-parallel β-sheet content is then calculated using the equivalent of eq. S3 above, where the sum runs over all potential anti-parallel β-sheets. Finally, the CV is computed by summing the total parallel and anti-parallel β-sheet contents. A Gaussian width equal to 0.33 was used for this CV.

3) The radius of gyration calculated on the Cα carbons, using the GYRATION keyword in PLUMED. This CV is defined as

$$s_{\mathrm{Gyr}} = \sqrt{\frac{\sum_{i\epsilon \mathrm{C}_\alpha} m_i |r_i\text{-}r_{\mathrm{COM}}|^2}{\sum_{i\epsilon \mathrm{C}_\alpha}^{\mathrm{n}} m_i}} \quad \text{(S4)}$$

where m_i and r_i and are the mass and position of the Cα atom of the ith residue, respectively and r_{COM} is the coordinate of the center of mass defined as

$$r_{\mathrm{COM}} = \frac{\sum_{i\epsilon \mathrm{C}_\alpha} m_i r_i}{\sum_{i\epsilon \mathrm{C}_\alpha} m_i} \quad \text{(S5)}$$

A Gaussian width equal to 0.03 nm was used for this CV.

4) The number of contacts between hydrophobic residues. This CV calculates the number of inter-Cβ atom distances in Ala, Cys, Ile, Leu, Met, Phe, Pro, Trp, or Val residues lower than 0.6 nm. It was calculated using the COORDINATION keyword in PLUMED using the following switching function

$$s_{\mathrm{Coord}} = \sum_{i\epsilon A} \sum_{j\epsilon B} \frac{1-\left(\frac{d_{ij}}{\mathrm{d}_0}\right)^6}{1-\left(\frac{d_{ij}}{\mathrm{d}_0}\right)^{12}} \quad \text{(S6)}$$

Where $d_0 = 0.6$ nm, d_{ij} is the distance between atoms i and j, and A and B are the two groups of atoms between which contacts are calculated. For this specific example, A and B are the

same group and self-interactions are excluded. A Gaussian width equal to 0.69 was used for this CV.

5) The number of salt-bridges. This CV is calculated as the number of contacts within a 0.6 nm cut-off range between the atoms of -COO^- groups of aspartic or glutamic acids and atoms of the -NH_3^+ groups of lysines or the -$C(NH_2)_2^+$ of arginines. This CV is computed using the COORDINATION keyword in PLUMED as defined by eq. S6 above. A Gaussian width equal to 2.75 was used.

6) The correlation between consecutive ψ torsion angles. This CV was calculated using the DIHCOR keyword in PLUMED using the following equation:

$$s_{\mathrm{Tors}} = \frac{1}{2}\sum_i [1 + \cos(\psi_i - \psi_{i+1})] \qquad \text{(S7)}$$

for every residue, i. A Gaussian width equal to 1.34 was used for this CV.

14 CVs were added to the bound simulation to enhance the sampling of contacts between atoms from the 10074-G5 molecule and atoms from consecutive three-residue regions of Aβ42. These were implemented using the COORDINATION keyword in PLUMED using the following equation:

$$s_{\mathrm{Con}} = \sum_{i\epsilon A}\sum_{j\epsilon B} \frac{1-\left(\frac{d_{ij}}{\mathrm{d}_0}\right)^n}{1-\left(\frac{d_{ij}}{\mathrm{d}_0}\right)^m} \qquad \text{(S8)}$$

where d_{ij} is the distance between atoms i and j, $n = 6$, $m = 12$, and $d_0 = 1.0$ Å. A Gaussian width of 1.0 was used for each of these CVs.

Lastly, 4 CVs were added to enhance the sampling of soft dihedrals of 10074-G5 using the TORSIONS keyword in PLUMED. A Gaussian width of 0.1 was used for each of these CVs. These were applied to the dihedrals in the top four subplots shown in fig. S4.

Details of structural ensemble analysis. The unbound and bound simulations contain 278,064 and 256,128 frames respectively. Prior to analysis, we checked that no frames in which the peptide interacted with its periodic image, such that the minimum distance between two peptides was less than 1.2 nm, were included. Furthermore, approximately the first 14 μs of the concatenated

trajectories were discarded for equilibration. For the bound ensemble, only frames in which 10074-G5 was in close contact with the peptide were considered, such that the minimum distance between the peptide and 10074-G5 was less than 0.8 nm (253,891 frames).

All observables are calculated as ensemble averages using the weights $w(\mathbf{s}_i)$ obtained from the bias potential $V_{\mathrm{PB}}(\mathbf{s}_i)$ at the end of metadynamics simulations (*73*) using

$$w(\mathbf{s}_i) = \frac{e^{\frac{V_{\mathrm{PB}}(\mathbf{s}_i)}{k_{\mathrm{B}}T}}}{\sum_j^N e^{\frac{V_{\mathrm{PB}}(\mathbf{s}_j)}{k_{\mathrm{B}}T}}} \tag{S9}$$

where $\mathbf{s}_i$ is the value of all CVs at time i, N is the total number of time steps, k_{B} is the Boltzmann constant, and T is the temperature.

Contact maps were computed based on Lennard-Jones (LJ) and Coulomb interaction energies between residues calculated using the GROMACS gmx energy tool. Only short-range interaction energies were considered within a 1.2 nm cut-off. These interaction energies were then converted to a ternary matrix where in which interactions that were less than one standard deviation from the mean were assigned a value of -1, those within one standard deviation of the mean were assigned a value of 0, and those greater than one standard deviation of the mean were assigned a value of 1.

Hydrophobic surface areas were computed using the GROMACS gmx sasa tool. Hydrophobic atoms were defined as those whose partial charges range from -0.2 to 0.2, all other atoms were defined as hydrophilic. Relative hydrophobic surface areas were computed by normalising over the total solvent exposed surface area.

To characterize the structural similarities and differences between the unbound and bound simulations and to assess their convergence, we performed a cluster analysis of the trajectories (Fig. 3B to D). Given that the cluster analysis is highly memory-intensive, we sampled a subset of 35,000 frames from each trajectory based on the metadynamics weights. Inter-residue LJ interaction energies were calculated for each frame and the shuffled data used as input for the GROMOS clustering algorithm (*34*) using the root-mean-squared-deviation (RMSD) as a measure of similarity between matrices. We used cut-off values equal to 8.5 kJ mol^{-1} to identify clusters and determine their populations. To assess convergence of a given simulation, we calculated the standard deviations of the populations of these states between the first and second halves of the analyzed trajectories (Fig. 3C). To depict each

cluster graphically, 10 frames were randomly selected based on their distance to the cluster centre as defined by the clustering algorithm.

We computed the conformation entropy (S) of the peptide using:

$$S = -\sum p \ln p \quad \text{(S10)}$$

where p is the fractional occupancy of a given cluster identified using the GROMOS clustering analysis above. This was repeated for several cut-off values (Fig. 3D).

The LJ and Coulomb interaction energies between residues and 10074-G5 were computed using the GROMACS gmx energy tool (Fig. 2E). Only short-range interaction energies were considered. Convergence was analyzed by comparing values from the first and second halves of the analyzed trajectories.

Hydrogen bonds were calculated using the GROMACS gmx hbond tool. Solvent atoms were defined to be in the shell if they were within 0.4 nm of the protein and/or 10074-G5 (*32*). All other solvent atoms were considered to be part of the bulk. The number of hydrogen bonds in the shell was computed as the sum of 1) the number of hydrogen atoms between shell and protein atoms (H_{SP}), 2) the number of hydrogen atoms between shell and bulk atoms (H_{SB}), 3) two times the number of hydrogen atoms between all shell atoms ($2 \times H_{\text{SS}}$), and, 4) in the case of the holo simulations, between the shell and small molecule (H_{SM}). This number was then divided by one-third of the total number of atoms in the shell to obtain the average number of hydrogen bonds per water molecule in the shell. Similarly, the number of hydrogen bonds in the bulk was computed as the sum of 1) the number of hydrogen atoms between bulk and shell atoms (H_{BS}), 2) two times the number of hydrogen atoms between all bulk atoms ($2 \times H_{\text{BB}}$), and, 3) in the case of the holo simulations, between the bulk and small molecule (H_{SB}). This number was then divided by one-third of the total number of atoms in the bulk to obtain the average number of hydrogen bonds per water molecule in the bulk.

Kinetic analysis of experimental aggregation data using a monomer sequestration model. The kinetic traces of Aβ42 aggregation in the absence of the inhibitor are described by the following formula (*35*)

$$\frac{M(t)}{M(\infty)} = 1 - \left(\frac{B_+ + C_+}{B_+ + C_+ e^{\kappa t}} \frac{B_- + C_+ e^{\kappa t}}{B_- + C_+}\right)^{\frac{k_\infty^2}{\kappa \tilde{k}_\infty}} e^{-k_\infty t}, \quad \text{(S11a)}$$

where $M(t)$ is the concentration of monomer found within the fibrils (proportional to the ThT fluorescence signal), and (*35*)

$$C_{\pm} = \frac{\lambda^2}{2\kappa^2}, \tag{S11b}$$

$$k_{\infty} = \kappa\sqrt{\frac{2}{n_2(n_2+1)} + \frac{2\lambda^2}{n_c\kappa^2}}, \tag{S11c}$$

$$\tilde{k}_{\infty} = \sqrt{k_{\infty}^2 - 4C_+C_-\kappa^2}, \tag{S11d}$$

$$B_{\pm} = \frac{k_{\infty} \pm \tilde{k}_{\infty}}{2\kappa}, \tag{S11e}$$

$$\lambda = \sqrt{2k_+k_{\mathrm{n}}m(0)^{n_{\mathrm{c}}}}, \tag{S11f}$$

$$\kappa = \sqrt{2k_+k_2m(0)^{n_2+1}}. \tag{S11g}$$

Here, k_+, k_{n}, and k_2 are the rate constants for fibril elongation, primary nucleation, and secondary nucleation, respectively. $m(0)$ is the initial monomer mass concentration, and $M(\infty)$ is the fibril mass concentration at the end of the aggregation reaction. $M(\infty)$ is given explicitly by $M(\infty) = m(0) - m_{\mathrm{critical}}$, where $m_{\mathrm{critical}} = k_-/k_+$ is the critical monomer concentration and k_- is the rate constant of monomer dissociation from the fibril ends. The parameters $\mathrm{n_c} = 2$ and $n_2 = 2$ are the reaction orders of primary and secondary nucleation (*35*). We used eq. S11a to extract the rate parameters $k_{\mathrm{n}}k_+$ and k_2k_+ from a global fit of independent kinetic traces measured at increasing concentrations of Aβ42 in the absence of the compound (Fig. 5B). This fit of the unperturbed kinetic curve yields the following values for the combined rate parameters $k_nk_+ = 1.6 \times 10^2 \pm 0.08 \times 10^2\ \mathrm{M^{-2}s^{-2}}$ and $k_2k_+ = 5.9 \times 10^{10} \pm 0.1 \times 10^{10}\ \mathrm{M^{-3}s^{-2}}$, consistent with the previous analysis (*3*).

Next, we developed a monomer sequestration model to describe kinetic traces in the presence of increasing concentrations of the compound (Fig. 5A). In this model, we hypothesize that a reduction of the concentration of free monomers available for aggregation due to sequestration can account both for the observed delay in aggregation and the decreased fibril load, which are both observed in the data. This situation is captured by the following kinetic equations:

$$\frac{\mathrm{d}P(t)}{\mathrm{d}t} = k_{\mathrm{n}} m(t)^{\mathrm{n_c}} + k_2 m(t)^{\mathrm{n_2}} M(t), \tag{S12a}$$

$$\frac{\mathrm{d}m(t)}{\mathrm{d}t} = -(2k_+ m(t) - 2k_-)P(t) - k_{\mathrm{on}}[C]\, m(t) + k_{\mathrm{off}} m_{\mathrm{bound}}(t), \tag{S12b}$$

$$\frac{\mathrm{d}m_{\mathrm{bound}}(t)}{\mathrm{d}t} = k_{\mathrm{on}} m(t)[C] - k_{\mathrm{off}} m_{\mathrm{bound}}(t), \tag{S12c}$$

where $[C]$ is the concentration of the inhibitor 10074-G5, $m(t)$ and $m_{\mathrm{bound}}(t)$ are, respectively, the concentrations of free and bound monomers; moreover, k_{on} and k_{off} are the association and dissociation constants of the inhibitor to/from the monomers, respectively (*3*). We work in a regime where the binding and unbinding rates of the inhibitor are fast compared to the overall rate of aggregation κ. For inhibitor concentrations between 1 μM and 20 μM, the rate of inhibitor binding ranges from $k_{\mathrm{on}}[C] = 8.5 \times 10^{-3}$ to $1.7 \times 10^{-1}\ \mathrm{s}^{-1}$, while the rate of unbinding is $k_{\mathrm{off}} = 4.7 \times 10^{-2}\ \mathrm{s}^{-1}$. These rates are faster than that of the overall aggregation reaction $\kappa = 3.0 \times 10^{-4}\ \mathrm{s}^{-1}$ (for 1 μM Aβ42), as determined from a global fit of unperturbed aggregation traces to eq. S11 (Fig. 5B). Due to this separation of timescales, aggregation in the presence of the inhibitor occurs in two stages (fig. S8). First, a rapid pre-equilibrium is established between free and inhibitor-bound monomers, yielding a fast, initial drop in free monomer concentration and a rapid increase in bound monomer fraction (fig. S8, A and B). The values of free and bound monomer concentrations at the end this rapid pre-equilibrium phase correspond to an equilibrium between free and inhibitor-bound monomers are obtained by setting $\frac{\mathrm{d}m_{\mathrm{bound}}(t)}{\mathrm{d}t} = 0$ in eq. S12, yielding

$$\frac{m_{\mathrm{bound}}}{m(0)} = 1 - \frac{1}{1+[C]/K_{\mathrm{d}}} \quad \text{and} \quad \frac{m}{m(0)} = \frac{1}{1+[C]/K_{\mathrm{d}}}, \tag{S13}$$

where $\mathrm{K_d} = \mathrm{k_{off}}/\mathrm{k_{on}}$ is the affinity of 10074-G5 for the monomer. These expressions correspond to the dashed lines in fig. S8, A and B and enter as initial conditions for the second, slower phase of aggregation, determining in this manner the extent of inhibition. During the second phase of aggregation, fibrils form from an effectively lower monomer concentration, which is the origin of the observed retardation of aggregation and decrease in the final fibril load. The aggregation reaction proceeds until equilibrium is reached. The equilibrium concentrations of free and bound monomers are obtained by applying the principle of detailed balance. The equilibrium concentration of free monomers equals the critical monomer concentration, independently of the amount of inhibitor added. The equilibrium concentration of inhibitor-bound monomers is dependent on the inhibitor concentration and is from eq. S13 as $m_{\mathrm{bound}} = \frac{m_{\mathrm{critical}}[C]}{K_{\mathrm{d}}}$. Summing the critical monomer

concentration and the equilibrium concentration of monomers bound to 10074-G5 yields the following expression for the equilibrium concentration of unreacted soluble monomer left at the end of the aggregation reaction:

$$m_{\text{unreacted}} = m_{\text{critical}} + m_{\text{bound}} = m_{\text{critical}}\left(1 + \frac{[C]}{K_{\text{d}}}\right). \tag{S14}$$

We can also obtain an explicit integrated rate law describing the time course of aggregation in the presence of the inhibitor (fig. S8C). The explicit equation describing the time-varying aggregate mass concentration is found to be identical to eq. S11, but the parameters λ and κ are replaced by renormalized ones, given by

$$\frac{\lambda^{\text{eff}}}{\lambda} = \left(\frac{1}{1+[C]/K_{\text{d}}}\right)^{\frac{n_c+1}{2}} \tag{S15a}$$

and

$$\frac{\kappa^{\text{eff}}}{\kappa} = \left(\frac{1}{1+[C]/K_{\text{d}}}\right)^{\frac{n_2+1}{2}}. \tag{S15b}$$

Similarly, the effective rate of elongation depends on the compound concentration as:

$$\frac{{k_+}^{\text{eff}}}{k_+} = \frac{1}{1+[C]/K_{\text{d}}}. \tag{S15c}$$

The performance of our integrated rate law against numerical integration of the master equation (eq. S12) is shown in fig. S8C. We used these explicit expressions to fit globally kinetic traces in the presence of increasing concentrations of the compound. In particular, we used eq. S11, where the kinetic parameters vary with 10074-G5 concentration according to eq. S15. This global fit to the normalized data has one single fitting parameter K_{d}, yields $K_{\text{d}} = 40$ µM and is shown in Fig. 5A. To validate these fits, we applied this model to independent kinetic traces measured at increasing concentrations of Aβ42 in the presence of 10 µM of 10074-G5 (Fig. 5C). We used eq. S11 and eq. S15 with the same parameters as in Fig. 5A to predict the reduction of monomer concentration available for aggregation due to the presence of 10 µM of the inhibitor and predicted the resulting kinetic profiles without introducing any fitting parameters (Fig. 5C). This analysis was done in Mathematica Version 11.0.0.0 and using the online available Amylofit platform (*44*).

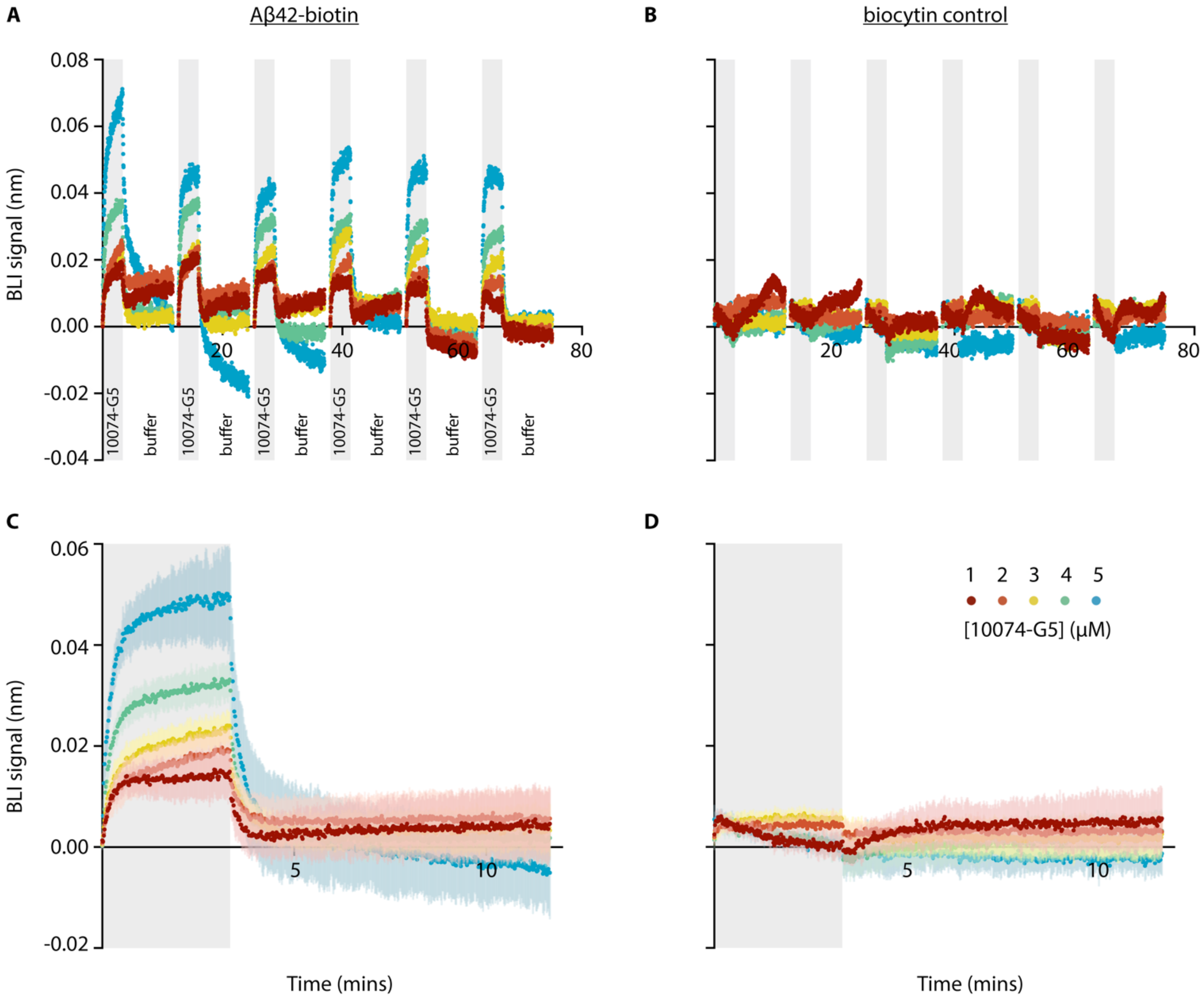

fig. S1. Biolayer interferometry (BLI) measurements showing the dose-dependent binding of 10074-G5 to Aβ42-fuctionalized and control surfaces. Real-time binding curves obtained from BLI super streptavidin sensor tips functionalized with Aβ42-biotin (**A**) or biocytin as a control for non-specific binding (**B**) after repeated exposure to 10074-G5 (grey) and buffer (white). All curves have been corrected for baseline drift. (**C, D**) Average BLI response (dots) for (A) and (B), respectively. Error bars correspond to ± SDs. 10074-G5 concentrations are shown in (D).

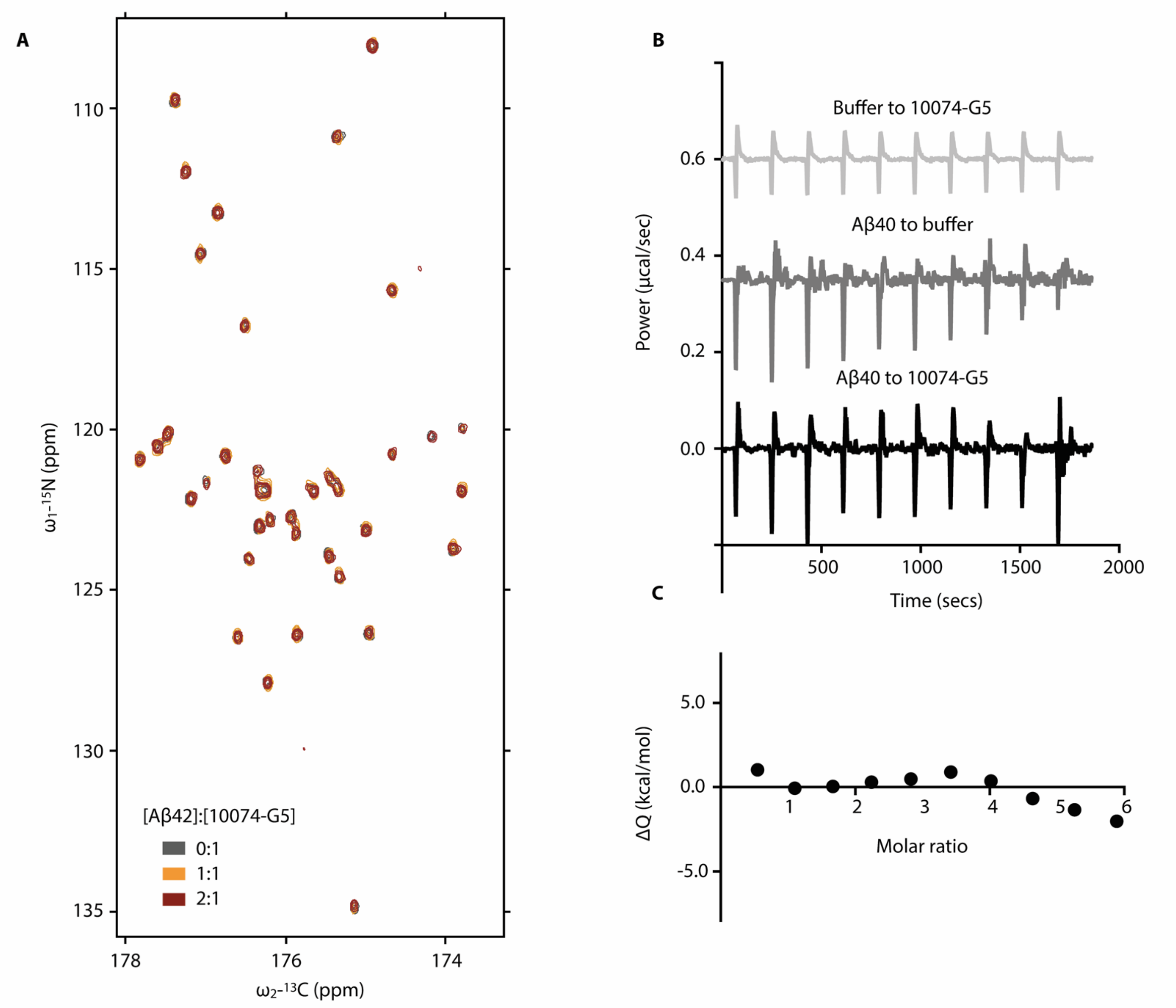

fig. S2. Characterization of the interaction of 10074-G5 with Aβ using NMR and isothermal titration calorimetry. (**A**) 2D H$^{N\text{–}BEST}$CON spectra of Aβ42 in the absence (grey) and presence of 1- (orange) 2-(red) fold concentration of 10074-G5 at 5 °C. (**B**) Isothermal titration calorimetry experiment of monomeric Aβ40 (200 µM) titrated into a solution of 7 µM 10074-G5 with corresponding heats of dilution. Dilution controls are shifted vertically for visibility. (**C**) Integrated peaks from (B) accounting for heats of dilution.

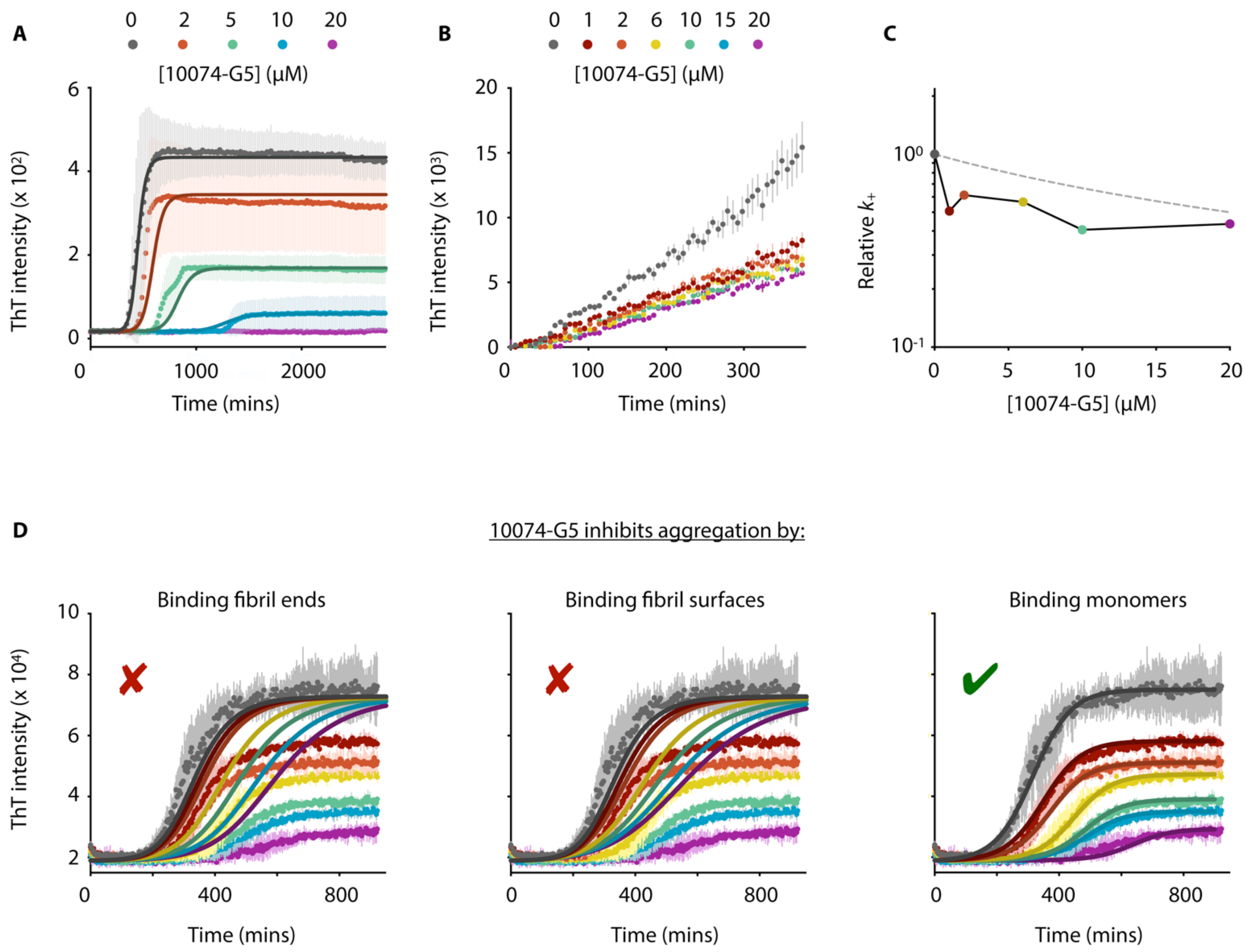

fig. S3. 10074-G5 inhibits Aβ40 aggregation and effects Aβ42 elongation. (**A**) 10074-G5 inhibits Aβ40 aggregation in an in vitro assay. ThT kinetic traces (see "ThT aggregation kinetics" in Materials and Methods) of the aggregation of 10 μM Aβ40 at increasing concentrations of 10074-G5. Measurements were taken in quintuplicate. Solid lines show global fits to the monomer sequestration model (eq. S12), in which 10074-G5 affects the aggregation by binding free monomers. The unperturbed rate parameters were obtained by fitting the data without inhibitor to eq. S11. Kinetic curves in the presence of increasing inhibitor were then globally fit to eq. S11 with the inhibitor dependence of the perturbed rate parameters given by eq. S15, leaving K_d as the only global fitting parameter. The global fit yields K_d = 10 μM. (**B**, **C**) Measurement of the effects of 10074-G5 on the fibril elongation rate of Aβ42**.** (**B**) ThT aggregation experiments performed in the presence of 15% preformed fibrils in the presence and absence of varying concentrations of 10074-G5. Measurements were taken in triplicate. (**C**) Change of the elongation constant, k_+, as a function of the concentration of 10074-G5, determined from the data shown in panel (B). Grey dashed line represents the

theoretical prediction of effective elongation rates predicted from eq. S15c. (**D**) Given the data, including the dot blot analysis (Fig. 4C to E), AFM measurements (fig. S7), and measurements on stabilized oligomers (fig. S10), we considered the following scenarios to model the inhibition of Aβ42 aggregation (Fig. 4A): 10074-G5 inhibits aggregation either by binding fibril ends (left), binding fibril surfaces (middle) and binding the monomers (right). The only scenario that is consistent with retardation with delay and a decrease in plateau is inhibition by binding monomers. Colors correspond to those shown in (B). Error bars represent ± SDs in (A, B, D).

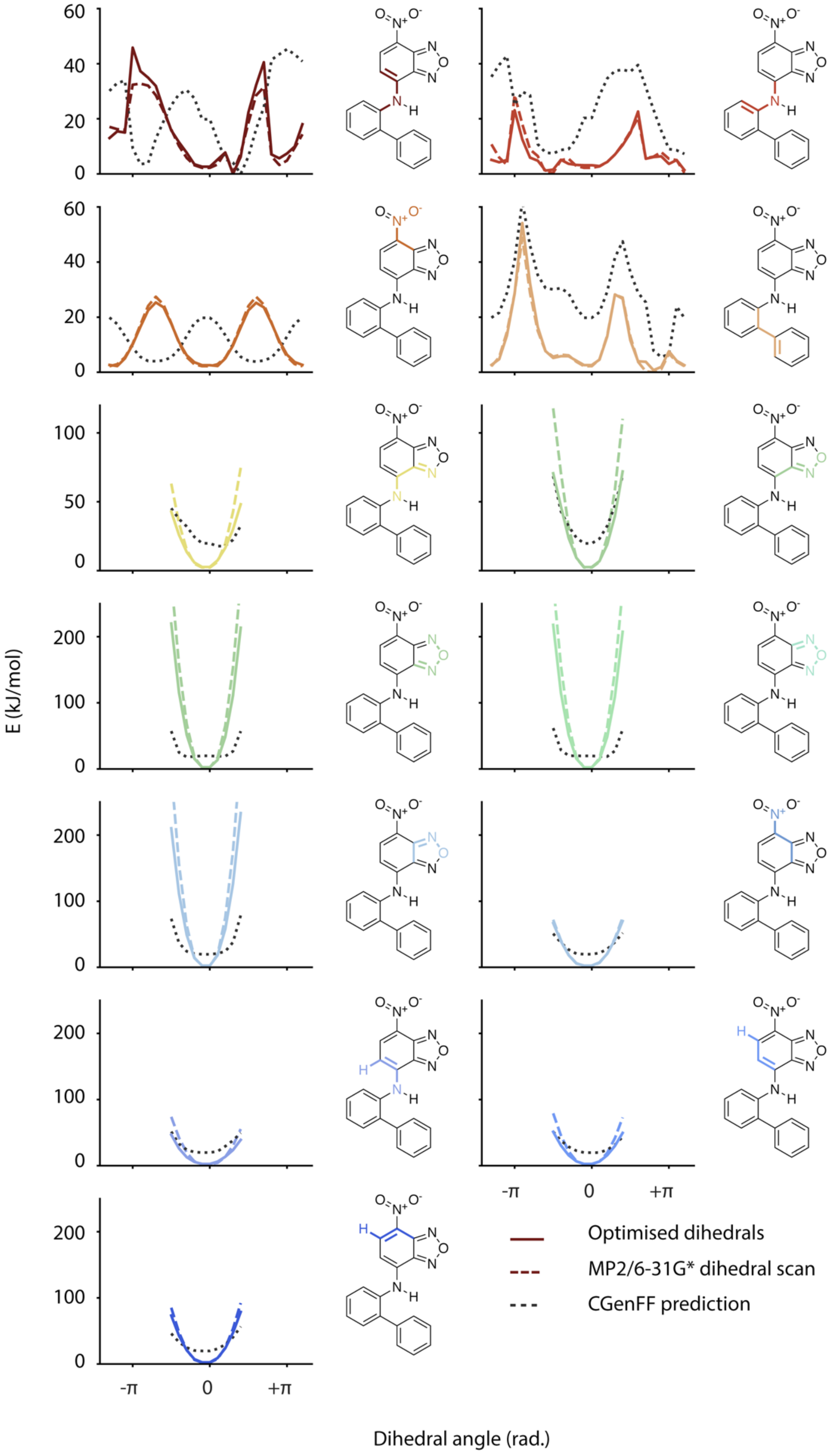

fig. S4. Parameterization of the dihedral angles of 10074-G5. Black dashed lines show the results from the CHARMM general force field (GGenFF) (*61*). Colored dashed lines show target data

obtained from quantum mechanical dihedral scans performed at the MP2/6-31G* level of theory (see "Details of 10074-G5 parameterization" above). Solid colored lines show dihedrals after optimization with the force field toolkit (*62*). Colored atoms and bonds on structures highlight the dihedral of interest for each fit.

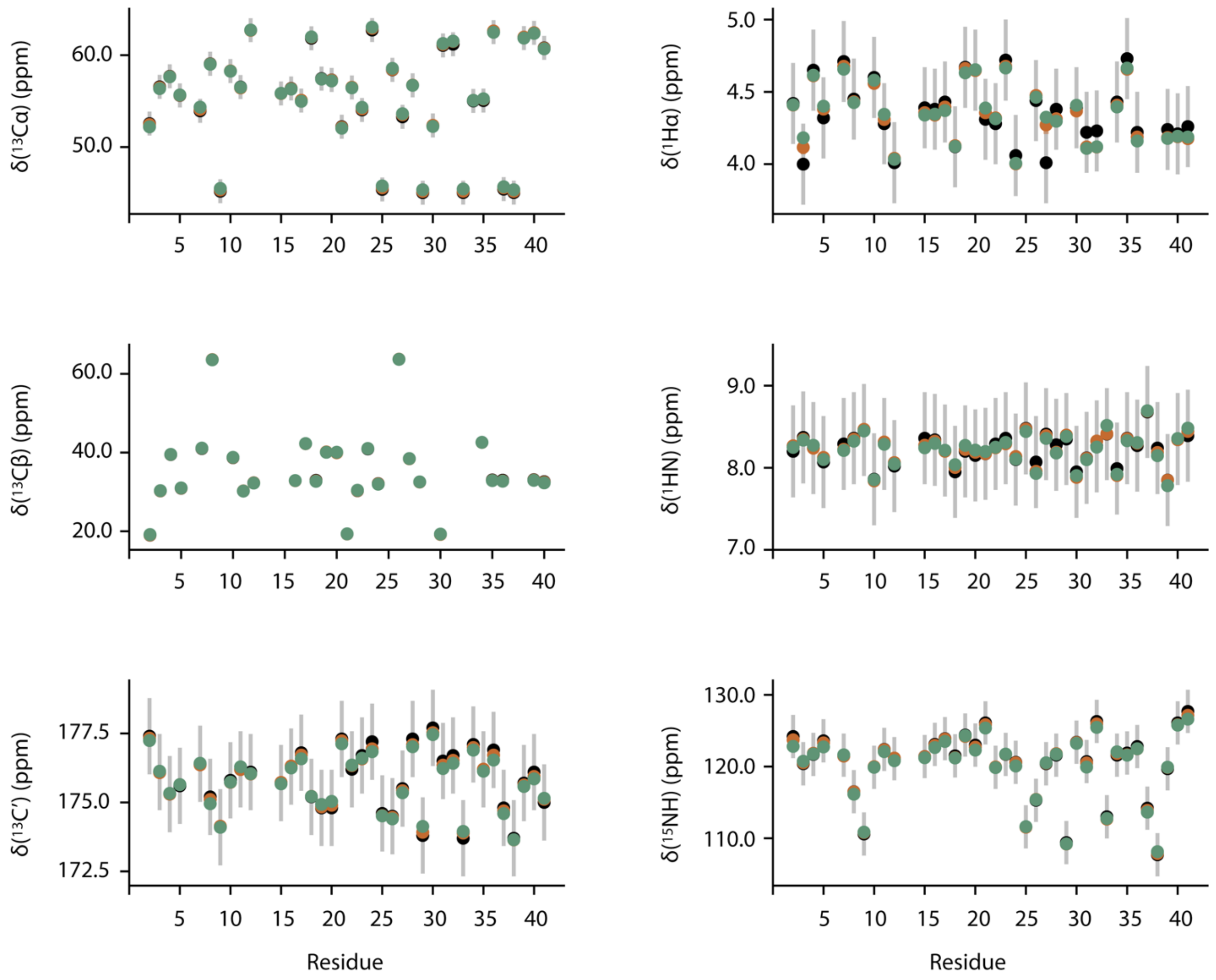

fig. S5. Assessment of the metadynamic metainference simulations using NMR chemical shifts. Comparison of the chemical shifts (δ) measured by NMR spectroscopy (black) with those predicted from the unbound (orange) and bound (green) simulations. NMR chemical shift data was used to restrain the simulations to improve force field accuracy. Grey error bars represent the errors associated with the CamShift predictor (*63*). Orange and green error bars for the unbound and bound simulations, respectively (sometimes smaller than data points) represent ± SDs between the chemical shifts calculated on the first and second halves of the analyzed trajectories.

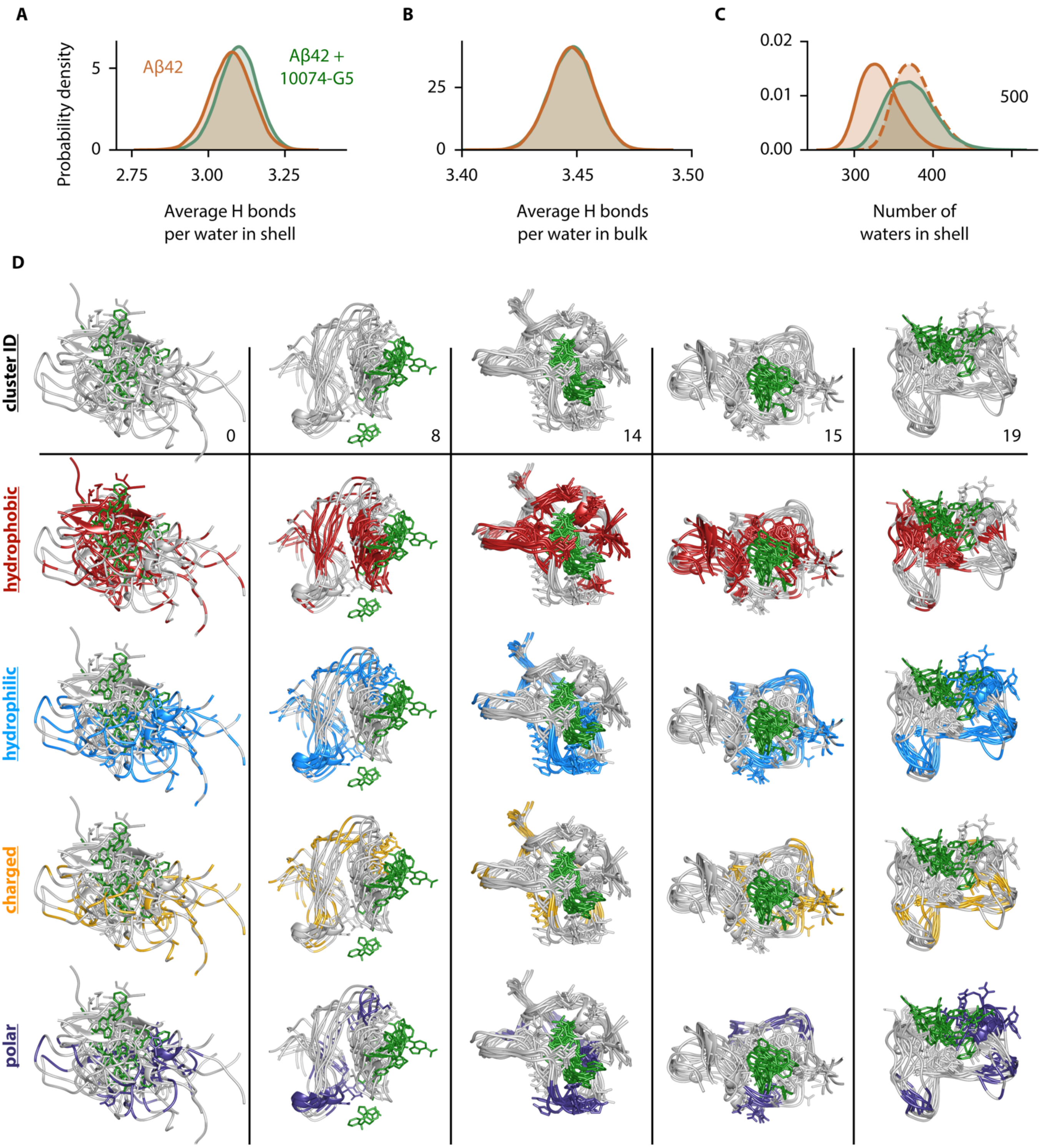

fig. S6. Characterization of water properties and illustration of key groups of residues involved in bound states. (**A** to **C**) Characterization of water properties in metadynamic metainference simulations. (**A**) Average number of hydrogen (H) bonds per water molecule in the shell (all water molecules within 0.4 nm of Aβ42 with and without 10074-G5). Distributions are shown using kernel density estimates of 35,000 points each sampled based on metadynamics weights using a Gaussian kernel. The orange curve represents the apo ensemble, the green curve represents the holo ensemble.

(**B**) Average number of H bonds per water molecule in the bulk (all water molecules not in the shell) using the same parameters and colors as shown in panel (A). (**C**) Kernel density estimates of the number of water molecule in the shell for the apo (orange curve, solid line) and holo (green curve, solid line) simulations. Using the dissociated frames from the holo simulation (in which 10074-G5 was further than 0.8 nm from the protein), we estimate the number of water molecules in the shell of 10074-G5 alone to be approximately 44.2. The orange dashed distribution represents the apo simulation shifted by this value. (**D**) Illustration depicting key groups of residues for heterogeneous bound clusters of Aβ42. Depiction of several conformations of Aβ42 (grey) with 10074-G5 (green) showing that many types of residues are involved in the binding interaction. Hydrophobic residues (red) include Ala, Val, Ile, Leu, Met, Phe, and Tyr. Hydrophilic residues (blue) include His, Ser, Asn, Gln, Tyr, Arg, Asp, Lys, and Glu. Charged residues (yellow) include Arg, Asp, Lys, and Glu. Polar residues (indigo) include His, Ser, Asn, Gln, and Tyr.

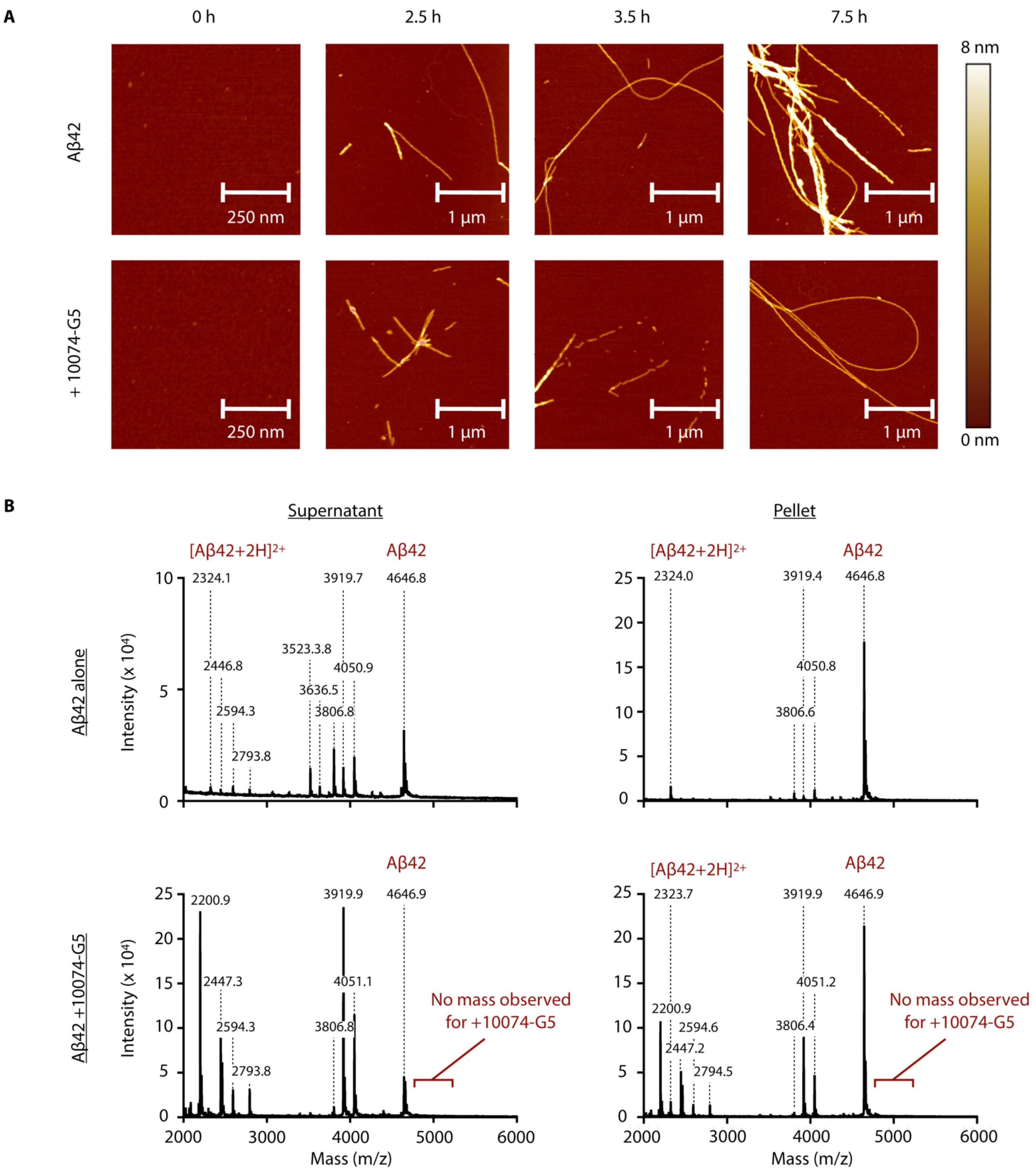

fig. S7. Further characterization of Aβ42 fibril formation in the presence and absence of 10074-G5. (**A**) AFM measurements show that 10074-G5 slows the formation of Aβ42 aggregation. Representative 3-D morphology maps from AFM time course experiments showing fibrils formed from 1 µM Aβ42 in the presence and absence of 6 µM 10074-G5. Single-molecule statistical analyses of these maps are shown in Fig. 4B. (**B**) Matrix assisted laser desorption/ionization (MALDI) mass spectrometry of Aβ42 in the presence and absence of 10074-G5. 15 µM of monomeric Aβ42 was

aggregated overnight at 37 °C in the absence (top) and presence (bottom) of 10074-G5. Samples were spun down to separate the supernatant (left) and pellet (right) (see Materials and Methods). MALDI mass spectrometry shows no chemical modification of Aβ42 by the presence of 10074-G5.

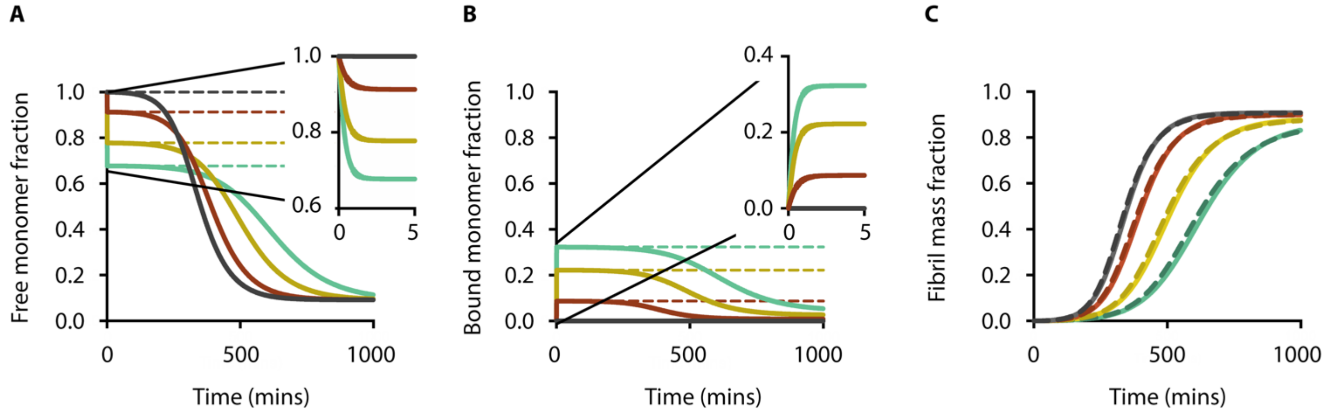

fig. S8. Behavior of solution to monomer sequestration model (eq. S11). (**A**) The time evolution of the free monomer concentration shows an initial rapid phase of pre-equilibration to eq. S13 (dashed lines), followed by slower aggregation ending at the critical concentration. (**B**) The time evolution of the concentration of bound monomers also displays two phases: a rapid pre-equilibration to eq. S13 and slow aggregation ending at eq. S14. (**C**) Time evolution of aggregate mass concentration is described by eq. S11 with effective parameters from eq. S15. A comparison between analytical (dashed lines) and numerical (solid lines) solution to the monomer sequestration model (eq. S12) shows excellent agreement.

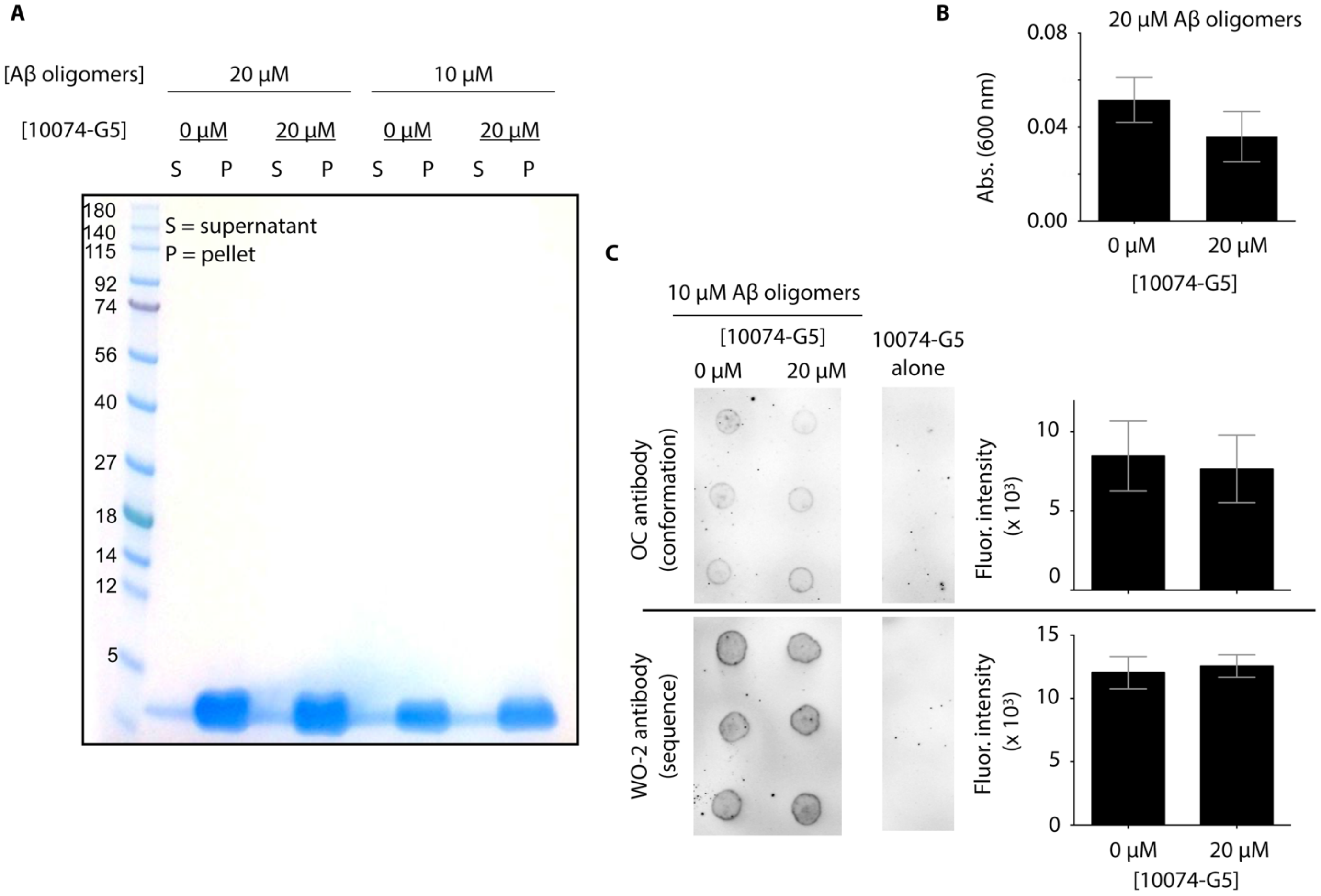

fig. S9. 10074-G5 does not alter pre-formed Aβ40 oligomers. (**A**) SDS gel of samples containing pre-formed Zn^{2+}-stabilized Aβ40 oligomers (20 μM and 10 μM) after incubation in the presence and absence of 20 μM 10074-G5. Samples were centrifuged to identify whether oligomers were dissociated into monomer upon addition of the compound. These data demonstrate that the compound does not dissociate pre-formed oligomers. (**B**) Turbidimetry measurements of 20 μM pre-formed Aβ40 oligomers show no significant difference in the presence of the compound, suggesting that 10074-G5 causes neither dissociation nor clustering of these oligomers. Measurements were background subtracted from buffers in the presence and absence of the compound, respectively. Measurements were taken in triplicate. (**C**) Dot blot using the OC-antibody shows that pre-formed oligomers maintain their cross β-sheet content in the presence of the 10074-G5. Measurements were taken in triplicate, as shown. Error bars represent ± SDs in (B, C).

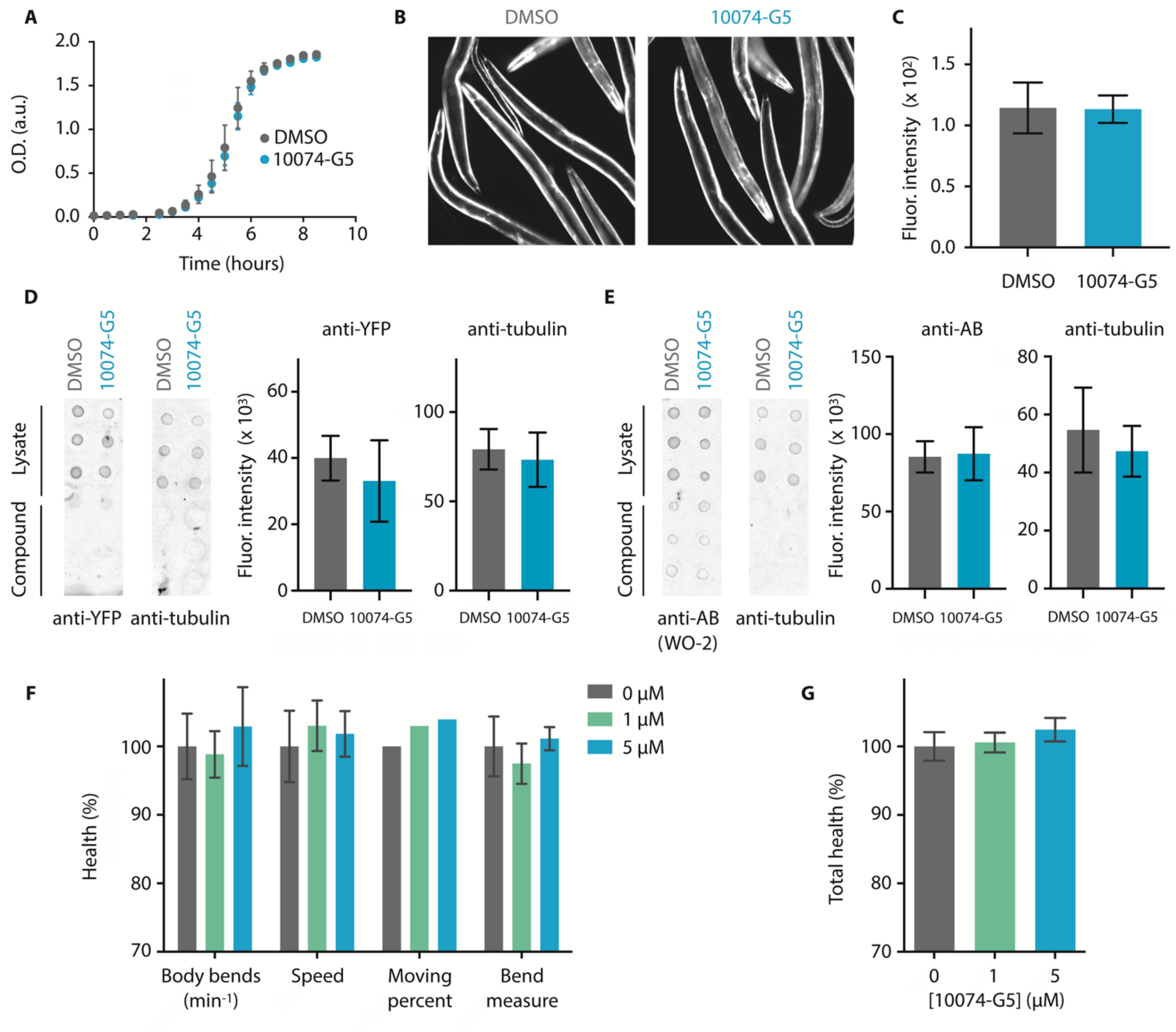

fig. S10. Further characterization of 10074-G5 and *C elegans*. (**A**) 10074-G5 does not alter the growth of *E. coli* strain OP50. Optical density (O.D.) growth curves of *E. coli* strain OP50 in the presence (blue) and absence of (grey) of 5 µM 10074-G5, showing that 10074-G5 does not alter the *E. coli* consumed by the *C. elegans*. (**B** to **E**) 10074-G5 does not alter the function of the unc-54 gene. (**B**) Representative fluorescence images of the OW450 *C. elegans* strain (rmIs126 [P(unc-54)Q0::YFP]V) with and without treatment of 5 µM 10074-G5 (Fig. 6A). (**C**) Quantification of over 100 worms in each condition shows no difference in the fluorescence of the yellow fluorescent protein (YFP), under control of the unc-54, with and without treatment. Dot-blots quantifying the levels of YFP in OW450 (**D**) and Aβ in the GMC101 *C. elegans* lysates (**E**). Immunodetection of α-tubulin was used as a control to ensure consistent loading of material (see Materials and Methods). Over 1000 and 5000 animals were analyzed for the OW450 and GMC101 strains, per condition, respectively. Measurements were taken in triplicate, as shown, error bars represent ± SDs in (D, E). (**F**, **G**) Effects

of 10074-G5 on wild-type *C. elegans*. (**F**) Health scores (%) for the rate of body bends, the speed of movement, the moving percent, and the magnitude of body bends at day 6 of adulthood. Error bars represent $\pm$ SEM, $n = 150$. (**G**) Combined total health scores from panel (F). Error bars represent $\pm$ SEM.

REFERENCES AND NOTES

1. J. Cummings, G. Lee, A. Ritter, K. Zhong, Alzheimer's disease drug development pipeline: 2019. *Alzheimers Dement.* **5**, 272–293 (2019).

2. T. P. Knowles, M. Vendruscolo, C. M. Dobson, The amyloid state and its association with protein misfolding diseases. *Nat. Rev. Mol. Cell Biol.* **15**, 384–396 (2014).

3. S. I. A. Cohen, S. Linse, L. M. Luheshi, E. Hellstrand, D. A. White, L. Rajah, D. E. Otzen, M. Vendruscolo, C. M. Dobson, T. P. J. Knowles, Proliferation of amyloid-β42 aggregates occurs through a secondary nucleation mechanism. *Proc. Natl. Acad. Sci. U.S.A.* **110**, 9758–9763 (2013).

4. J. Hardy, D. J. Selkoe, The amyloid hypothesis of Alzheimer's disease: Progress and problems on the road to therapeutics. *Science* **297**, 353–356 (2002).

5. C. Haass, D. J. Selkoe, Soluble protein oligomers in neurodegeneration: Lessons from the Alzheimer's amyloid β-peptide. *Nat. Rev. Mol. Cell Biol.* **8**, 101–112 (2007).

6. I. Benilova, E. Karran, B. De Strooper, The toxic Aβ oligomer and Alzheimer's disease: An emperor in need of clothes. *Nat. Neurosci.* **15**, 349–357 (2012).

7. P. Arosio, T. C. T. Michaels, S. Linse, C. Månsson, C. Emanuelsson, J. Presto, J. Johansson, M. Vendruscolo, C. M. Dobson, T. P. J. Knowles, Kinetic analysis reveals the diversity of microscopic mechanisms through which molecular chaperones suppress amyloid formation. *Nat. Commun.* **7**, 10948 (2016).

8. J. Habchi, S. Chia, R. Limbocker, B. Mannini, M. Ahn, M. Perni, O. Hansson, P. Arosio, J. R. Kumita, P. K. Challa, S. I. A. Cohen, S. Linse, C. M. Dobson, T. P. J. Knowles, M. Vendruscolo, Systematic development of small molecules to inhibit specific microscopic steps of Aβ42 aggregation in Alzheimer's disease. *Proc. Natl. Acad. Sci. U.S.A.* **114**, E200–E208 (2017).

9. J. Habchi, P. Arosio, M. Perni, A. R. Costa, M. Yagi-Utsumi, P. Joshi, S. Chia, S. I. A. Cohen, M. B. D. Müller, S. Linse, E. A. A. Nollen, C. M. Dobson, T. P. J. Knowles, M. Vendruscolo, An anticancer

drug suppresses the primary nucleation reaction that initiates the production of the toxic Aβ42 aggregates linked with Alzheimer's disease. *Sci. Adv.* **2**, e1501244 (2016).

10. F. A. Aprile, P. Sormanni, M. Perni, P. Arosio, S. Linse, T. P. J. Knowles, C. M. Dobson, M. Vendruscolo, Selective targeting of primary and secondary nucleation pathways in Aβ42 aggregation using a rational antibody scanning method. *Sci. Adv.* **3**, e1700488 (2017).

11. S. I. A. Cohen, P. Arosio, J. Presto, F. R. Kurudenkandy, H. Biverstål, L. Dolfe, C. Dunning, X. Yang, B. Frohm, M. Vendruscolo, J. Johansson, C. M. Dobson, A. Fisahn, T. P. J. Knowles, S. Linse, A molecular chaperone breaks the catalytic cycle that generates toxic Aβ oligomers. *Nat. Struct. Mol. Biol.* **22**, 207–213 (2015).

12. W. Hoyer, C. Grönwall, A. Jonsson, S. Ståhl, T. Härd, Stabilization of a β-hairpin in monomeric Alzheimer's amyloid-β peptide inhibits amyloid formation. *Proc. Natl. Acad. Sci. U.S.A.* **105**, 5099–5104 (2008).

13. C. Lendel, B. Bolognesi, A. Wahlström, C. M. Dobson, A. Gräslund, Detergent-like interaction of Congo red with the amyloid β peptide. *Biochemistry* **49**, 1358–1360 (2010).

14. A. Abelein, B. Bolognesi, C. M. Dobson, A. Gräslund, C. Lendel, Hydrophobicity and conformational change as mechanistic determinants for nonspecific modulators of amyloid β self-assembly. *Biochemistry* **51**, 126–137 (2012).

15. P. Pratim Bose, U. Chatterjee, L. Xie, J. Johansson, E. Göthelid, P. I. Arvidsson, Effects of congo red on $A\beta_{1-40}$ fibril formation process and morphology. *ACS Chem. Nerosci.* **1**, 315–324 (2010).

16. B. Y. Feng, B. H. Toyama, H. Wille, D. W. Colby, S. R. Collins, B. C. H. May, S. B. Prusiner, J. Weissman, B. K. Shoichet, Small-molecule aggregates inhibit amyloid polymerization. *Nat. Chem. Biol.* **4**, 197–199 (2008).

17. J. Habchi, P. Tompa, S. Longhi, V. N. Uversky, Introducing protein intrinsic disorder. *Chem. Rev.* **114**, 6561–6588 (2014).

18. G. T. Heller, P. Sormanni, M. Vendruscolo, Targeting disordered proteins with small molecules using entropy. *Trends Biochem. Sci.* **40**, 491–496 (2015).

19. G. T. Heller, M. Bonomi, M. Vendruscolo, Structural ensemble modulation upon small molecule binding to disordered proteins. *J. Mol. Biol.* **430**, 2288–2292(2018).

20. V. Csizmok, A. V. Follis, R. W. Kriwacki, J. D. Forman-Kay, Dynamic protein interaction networks and new structural paradigms in signaling. *Chem. Rev.* **116**, 6424–6462 (2016).

21. X. Yin, C. Giap, J. S. Lazo, E. V. Prochownik, Low molecular weight inhibitors of Myc–Max interaction and function. *Oncogene* **22**, 6151–6159 (2003).

22. A. V. Follis, D. I. Hammoudeh, H. Wang, E. V. Prochownik, S. J. Metallo, Structural rationale for the coupled binding and unfolding of the c-Myc oncoprotein by small molecules. *Chem. Biol.* **15**, 1149–1155 (2008).

23. D. M. Clausen, J. Guo, R. A. Parise, J. H. Beumer, M. J. Egorin, J. S. Lazo, E. V. Prochownik, J. L. Eiseman, In vitro cytotoxicity and in vivo efficacy, pharmacokinetics, and metabolism of 10074-G5, a novel small-molecule inhibitor of c-Myc/Max dimerization. *J. Pharmacol. Exp. Ther.* **335**, 715–727 (2010).

24. M. A. Reed *et al.* (Google Patents, 2018).

25. S. Gil, T. Hošek, Z. Solyom, R. Kümmerle, B. Brutscher, R. Pierattelli, I. C. Felli, NMR spectroscopic studies of intrinsically disordered proteins at near-physiological conditions. *Angew. Chem. Int. Ed. Engl.* **52**, 11808–11812 (2013).

26. M. Bonomi, G. T. Heller, C. Camilloni, M. Vendruscolo, Principles of protein structural ensemble determination. *Curr. Opin. Struct. Biol.* **42**, 106–116 (2017).

27. G. T. Heller, F. A. Aprile, M. Bonomi, C. Camilloni, A. de Simone, M. Vendruscolo, Sequence specificity in the entropy-driven binding of a small molecule and a disordered peptide. *J. Mol. Biol.* **429**, 2772–2779 (2017).

28. M. Bonomi, C. Camilloni, M. Vendruscolo, Metadynamic metainference: Enhanced sampling of the metainference ensemble using metadynamics. *Sci. Rep.* **6**, 31232 (2016).

29. M. Bonomi, C. Camilloni, A. Cavalli, M. Vendruscolo, Metainference: A Bayesian inference method for heterogeneous systems. *Sci. Adv.* **2**, e1501177 (2016).

30. M. Fuxreiter, Fold or not to fold upon binding—Does it really matter? *Curr. Opin. Struct. Biol.* **54**, 19–25 (2019).

31. M. Friedemann, E. Helk, A. Tiiman, K. Zovo, P. Palumaa, V. Tõugu, Effect of methionine-35 oxidation on the aggregation of amyloid-β peptide. *Biochem. Biophys. Rep.* **3**, 94–99 (2015).

32. C. Camilloni, D. Bonetti, A. Morrone, R. Giri, C. M. Dobson, M. Brunori, S. Gianni, M. Vendruscolo, Towards a structural biology of the hydrophobic effect in protein folding. *Sci. Rep.* **6**, 28285 (2016).

33. V. M. Burger, D. J. Arenas, C. M. Stultz, A structure-free method for quantifying conformational flexibility in proteins. *Sci. Rep.* **6**, 29040 (2016).

34. X. Daura, K. Gademann, B. Jaun, D. Seebach, W. F. van Gunsteren, A. E. Mark, Peptide folding: When simulation meets experiment. *Angew. Chem. Int. Ed. Engl.* **38**, 236–240 (1999).

35. S. I. Cohen, M. Vendruscolo, C. M. Dobson, T. P. Knowles, Nucleated polymerization with secondary pathways. II. Determination of self-consistent solutions to growth processes described by non-linear master equations. *J. Chem. Phys.* **135**, 0656106 (2011).

36. T. P. J. Knowles, C. A. Waudby, G. L. Devlin, S. I. A. Cohen, A. Aguzzi, M. Vendruscolo, E. M. Terentjev, M. E. Welland, C. M. Dobson, An analytical solution to the kinetics of breakable filament assembly. *Science* **326**, 1533–1537 (2009).

37. D. M. Walsh, E. Thulin, A. M. Minogue, N. Gustavsson, E. Pang, D. B. Teplow, S. Linse, A facile method for expression and purification of the Alzheimer's disease-associated amyloid β-peptide. *FEBS J.* **276**, 1266–1281 (2009).

38. P. Arosio, M. Vendruscolo, C. M. Dobson, T. P. J. Knowles, Chemical kinetics for drug discovery to combat protein aggregation diseases. *Trends Pharmacol. Sci.* **35**, 127–135 (2014).

39. O. J. Rolinski, M. Amaro, D. J. Birch, Early detection of amyloid aggregation using intrinsic fluorescence. *Biosens. Bioelectron.* **25**, 2249–2252 (2010).

40. S. De, D. C. Wirthensohn, P. Flagmeier, C. Hughes, F. A. Aprile, F. S. Ruggeri, D. R. Whiten, D. Emin, Z. Xia, J. A. Varela, P. Sormanni, F. Kundel, T. P. J. Knowles, C. M. Dobson, C. Bryant, M. Vendruscolo, D. Klenerman, Different soluble aggregates of Aβ42 can give rise to cellular toxicity through different mechanisms. *Nat. Commun.* **10**, 1541 (2019).

41. F. S. Ruggeri, S. Vieweg, U. Cendrowska, G. Longo, A. Chiki, H. A. Lashuel, G. Dietler, Nanoscale studies link amyloid maturity with polyglutamine diseases onset. *Sci. Rep.* **6**, 31155 (2016).

42. F. S. Ruggeri, J. Adamcik, J. S. Jeong, H. A. Lashuel, R. Mezzenga, G. Dietler, Influence of the β-sheet content on the mechanical properties of aggregates during amyloid fibrillization. *Angew. Chem. Int. Ed. Engl.* **54**, 2462–2466 (2015).

43. F. S. Ruggeri, F. Benedetti, T. P. J. Knowles, H. A. Lashuel, S. Sekatskii, G. Dietler, Identification and nanomechanical characterization of the fundamental single-strand protofilaments of amyloid α-synuclein fibrils. *Proc. Natl. Acad. Sci. U.S.A.* **115**, 7230–7235 (2018).

44. G. Meisl, J. B. Kirkegaard, P. Arosio, T. C. T. Michaels, M. Vendruscolo, C. M. Dobson, S. Linse, T. P. J. Knowles, Molecular mechanisms of protein aggregation from global fitting of kinetic models. *Nat. Protoc.* **11**, 252–272 (2016).

45. T. C. T. Michaels, G. Meisl, G. T. Heller, S. Curk, P. Arosio, S. Linse, C. M. Dobson, M. Vendruscolo, T. P. J. Knowles, Thermodynamic and kinetic design principles for protein aggregation inhibitors. *bioRxiv* 10.1101/2020.02.22.960716. 20 February 2020.

46. B. Mannini, J. Habchi, S. Chia, F. S. Ruggeri, M. Perni, T. P. J. Knowles, C. M. Dobson, M. Vendruscolo, Stabilization and characterization of cytotoxic $A\beta_{40}$ oligomers isolated from an aggregation reaction in the presence of zinc ions. *ACS Chem. Nerosci.* **9**, 2959–2971 (2018).

47. R. Kayed, E. Head, F. Sarsoza, T. Saing, C. W. Cotman, M. Necula, L. Margol, J. Wu, L. Breydo, J. L. Thompson, S. Rasool, T. Gurlo, P. Butler, C. G. Glabe, Fibril specific, conformation dependent antibodies recognize a generic epitope common to amyloid fibrils and fibrillar oligomers that is absent in prefibrillar oligomers. *Mol. Neurodegener.* **2**, 18 (2007).

48. G. McColl, B. R. Roberts, T. L. Pukala, V. B. Kenche, C. M. Roberts, C. D. Link, T. M. Ryan, C. L. Masters, K. J. Barnham, A. I. Bush, R. A. Cherny, Utility of an improved model of amyloid-beta ($A\beta_{1-42}$) toxicity in *Caenorhabditis elegans* for drug screening for Alzheimer's disease. *Mol. Neurodegener.* **7**, 57 (2012).

49. S. Brenner, The genetics of *Caenorhabditis elegans*. *Genetics* **77**, 71–94 (1974).

50. T. J. Van Ham, K. L. Thijssen, R. Breitling, R. M. W. Hofstra, R. H. A. Plasterk, E. A. A. Nollen, *C. elegans* model identifies genetic modifiers of α-synuclein inclusion formation during aging. *PLOS Genet* **4**, e1000027 (2008).

51. E. Lescop, P. Schanda, B. Brutscher, A set of BEST triple-resonance experiments for time-optimized protein resonance assignment. *J. Magn. Reson.* **187**, 163–169 (2007).

52. M. J. Abraham, T. Murtola, R. Schulz, S. Páll, J. C. Smith, B. Hess, E. Lindahl, GROMACS: High performance molecular simulations through multi-level parallelism from laptops to supercomputers. *SoftwareX* **1–2**, 19–25 (2015).

53. PLUMED consortium, Promoting transparency and reproducibility in enhanced molecular simulations. *Nat. Methods* **16**, 670–673 (2019).

54. S. Piana, K. Lindorff-Larsen, D. E. Shaw, How robust are protein folding simulations with respect to force field parameterization? *Biophys. J.* **100**, L47–L49 (2011).

55. A. D. MacKerell Jr., D. Bashford, M. Bellott, R. L. Dunbrack Jr, J. D. Evanseck, M. J. Field, S. Fischer, J. Gao, H. Guo, S. Ha, D. Joseph-McCarthy, L. Kuchnir, K. Kuczera, F. T. K. Lau, C. Mattos, S. Michnick, T. Ngo, D. T. Nguyen, B. Prodhom, W. E. Reiher, B. Roux, M. Schlenkrich, J. C. Smith, R. Stote, J. Straub, M. Watanabe, J. Wiórkiewicz-Kuczera, D. Yin, M. Karplus, All-atom

empirical potential for molecular modeling and dynamics studies of proteins. *J. Phys. Chem. B* **102**, 3586–3616 (1998).

56. G. Bussi, D. Donadio, M. Parrinello, Canonical sampling through velocity rescaling. *J. Chem. Phys.* **126**, 014101 (2007).

57. H. J. Berendsen, J. V. Postma, W. F. van Gunsteren, A. DiNola, J. Haak, Molecular dynamics with coupling to an external bath. *J. Chem. Phys.* **81**, 3684–3690 (1984).

58. M. Parrinello, A. Rahman, Polymorphic transitions in single crystals: A new molecular dynamics method. *J. Appl. Phys.* **52**, 7182–7190 (1981).

59. B. Hess, H. Bekker, H. J. C. Berendsen, J. G. E. M. Fraaije, LINCS: A linear constraint solver for molecular simulations. *J. Comput. Chem.* **18**, 1463–1472 (1997).

60. U. Essmann, L. Perera, M. L. Berkowitz, T. Darden, H. Lee, L. G. Pedersen, A smooth particle mesh Ewald method. *J. Chem. Phys.* **103**, 8577–8593 (1995).

61. K. Vanommeslaeghe, A. D. MacKerell Jr., Automation of the CHARMM General Force Field (CGenFF) I: Bond perception and atom typing. *J. Chem. Inf. Model.* **52**, 3144–3154 (2012).

62. C. G. Mayne, J. Saam, K. Schulten, E. Tajkhorshid, J. C. Gumbart, Rapid parameterization of small molecules using the force field toolkit. *J. Comput. Chem.* **34**, 2757–2770 (2013).

63. P. Robustelli, K. Kohlhoff, A. Cavalli, M. Vendruscolo, Using NMR chemical shifts as structural restraints in molecular dynamics simulations of proteins. *Structure* **18**, 923–933 (2010).

64. J. Pfaendtner, M. Bonomi, Efficient sampling of high-dimensional free-energy landscapes with Parallel Bias Metadynamics. *J. Chem. Theory Comput.* **11**, 5062–5067 (2015).

65. A. Barducci, G. Bussi, M. Parrinello, Well-tempered metadynamics: A smoothly converging and tunable free-energy method. *Phys. Rev. Lett.* **100**, (2008).

66. P. Raiteri, A. Laio, F. L. Gervasio, C. Micheletti, M. Parrinello, Efficient reconstruction of complex free energy landscapes by multiple walkers metadynamics. *J. Phys. Chem. B* **110**, 3533–3539 (2006).

67. M. Novo, S. Freire, W. Al-Soufi, Critical aggregation concentration for the formation of early Amyloid-β (1–42) oligomers. *Sci. Rep.* **8**, 1783 (2018).

68. M. Perni, C. Galvagnion, A. Maltsev, G. Meisl, M. B. D. Müller, P. K. Challa, J. B. Kirkegaard, P. Flagmeier, S. I. A. Cohen, R. Cascella, S. W. Chen, R. Limbocker, P. Sormanni, G. T. Heller, F. A. Aprile, N. Cremades, C. Cecchi, F. Chiti, E. A. A. Nollen, T. P. J. Knowles, M. Vendruscolo, A. Bax, M. Zasloff, C. M. Dobson, A natural product inhibits the initiation of α-synuclein aggregation and suppresses its toxicity. *Proc. Natl. Acad. Sci. U.S.A.* **114**, E1009-E1017 (2017).

69. M. Perni, P. K. Challa, J. B. Kirkegaard, R. Limbocker, M. Koopman, M. C. Hardenberg, P. Sormanni, T. Müller, K. L. Saar, L. W. Y. Roode, J. Habchi, G. Vecchi, N. Fernando, S. Casford, E. A. A. Nollen, M. Vendruscolo, C. M. Dobson, T. P. J. Knowles, Massively parallel *C. elegans* tracking provides multi-dimensional fingerprints for phenotypic discovery. *J. Neurosci. Methods* **306**, 57–67 (2018).

70. M. Perni, S. Casford, F. A. Aprile, E. A. Nollen, T. P. J. Knowles, M. Vendruscolo, C. M. Dobson, Automated behavioral analysis of large *C. elegans* populations using a wide field-of-view tracking platform. *J. Vis. Exp.*, e58643 (2018).

71. R. A. McCloy, S. Rogers, C. E. Caldon, T. Lorca, A. Castro, A. Burgess, Partial inhibition of Cdk1 in G_2 phase overrides the SAC and decouples mitotic events. *Cell Cycle* **13**, 1400–1412 (2014).

72. T. Löhr, A. Jussupow, C. Camilloni, Metadynamic metainference: Convergence towards force field independent structural ensembles of a disordered peptide. *J. Chem. Phys.* **146**, 165102 (2017).

73. D. Branduardi, G. Bussi, M. Parrinello, Metadynamics with adaptive Gaussians. *J. Chem. Theory Comput.* **8**, 2247–2254 (2012).
